# Inhibition of S6K lowers age-related inflammation and increases lifespan through the endolysosomal system

**DOI:** 10.1038/s43587-024-00578-3

**Published:** 2024-02-27

**Authors:** Pingze Zhang, James H. Catterson, Sebastian Grönke, Linda Partridge

**Affiliations:** 1 Max Planck Institute for Biology of Ageing, Cologne, Germany; 2 Institute of Healthy Ageing, Department of Genetics, Evolution and Environment, University College London, London, UK; 3 Present Address: Centre for Discovery Brain Sciences, UK Dementia Research Institute, University of Edinburgh, Edinburgh, UK

**Keywords:** Ageing, Inflammation

## Abstract

Suppression of target of rapamycin complex 1 (TORC1) by rapamycin ameliorates aging in diverse species. S6 kinase (S6K) is an essential mediator, but the mechanisms involved are unclear. Here we show that activation of S6K specifically in *Drosophila* fat-body blocked extension of lifespan by rapamycin, induced accumulation of multilamellar lysosomes and blocked age-associated hyperactivation of the NF-κB-like immune deficiency (IMD) pathway, indicative of reduced inflammaging. Syntaxin 13 mediated the effects of TORC1–S6K signaling on lysosome morphology and inflammaging, suggesting they may be linked. Inflammaging depended on the IMD receptor regulatory isoform PGRP-LC, and repression of the IMD pathway from midlife extended lifespan. Age-related inflammaging was higher in females than in males and was not lowered in males by rapamycin treatment or lowered S6K. Rapamycin treatment also elevated Syntaxin 12/13 levels in mouse liver and prevented age-related increase in noncanonical NF-κB signaling, suggesting that the effect of TORC1 on inflammaging is conserved from flies to mammals.

## Abstract (web summary)

Zhang et al. demonstrate that S6K suppression in the *Drosophila* fat body mediates the longevity effects of rapamycin and identify Syntaxin 13 as a conserved downstream effector regulating lysosome morphology and inflammation in a sex-specific manner.

## Main

The mammalian target of rapamycin (mTOR) network senses nutrients and various stressors and plays a key role in the regulation of growth, metabolism and longevity. mTOR is a serine/threonine protein kinase that is a crucial component of mTOR complex 1 (TORC1)^1^. Suppression of TORC1 signaling genetically or pharmacologically by the US Food and Drug Administration-licensed drug rapamycin (Rapa), promotes lifespan and healthspan in various model organisms including nematode worms, fruit flies and mice^2–8^.

Rapa increases lifespan in *Drosophila* via two evolutionarily conserved TORC1 downstream effector mechanisms: increased autophagy and reduced ribosomal S6 kinase (S6K) activity^3^. Autophagy is a lysosome-dependent cellular clearance pathway that promotes cellular and tissue homeostasis^9^. Promoting autophagy enhances longevity and suppresses age-related tissue deterioration in *Drosophila* and mice^9–12^. The intestine is a crucial organ mediating the effects of increased autophagy on health during aging^11–14^. S6K is an AGC family kinase that regulates fundamental cellular processes including translation, lipid metabolism and immunity^15^. Female but not male mice carrying a S6K1 null allele are long-lived indicating a sex-specific effect of S6K on lifespan regulation^16^. However, the mechanisms by which reduced S6K signaling promotes lifespan and healthspan remain elusive. *Drosophila* provides an ideal model organism to address this question, as it has only a single S6K, with considerable homology to the mammalian p70-S6K1 (ref. ^17^).

Rapa treatment also ameliorates aging-associated decline in immune function^18,19^. A chronic inflammation, termed ‘inflammaging’ and deterioration of immune function, termed ‘immunosenescence’^20,21^, both occur during aging. These contribute to a wide range of age-associated diseases, such as cancer, diabetes and cardiovascular diseases^21–23^. The nuclear factor-kappa B (NF-κB) pathway plays a key role in innate immunity, and Rapa treatment ameliorates senescence-associated NF-κB activation^24^. The effects of Rapa on inflammaging and lifespan are diminished in mice with genetically enhanced NF-κB activity (*nfκb1*^*−/−*^)^25^, suggesting that the late-life health-promoting effects of Rapa are mediated at least in part by limiting NF-κB signaling. Brief, late-life TORC1 inhibition can increase immune response of older people to influenza vaccination, without significant adverse effects^19^. Interestingly, TORC1 pathway activity shows a significant association with the load of bacteria in old flies^26,27^, suggesting that enhanced immune function from suppression of TORC1 may be conserved. However, the underlying molecular and cellular mechanisms of these TORC1-related effects are unclear.

In this Article, we identified the *Drosophila* fat body as an essential organ for suppression of S6K to extend lifespan. TORC1 inhibition by Rapa treatment repressed enlarged, multilamellar lysosomes in the *Drosophila* fat body, and this effect was blocked by elevating S6K activity. We identified Syntaxin 13 (Syx13), a SNARE family protein that mediates endomembrane function, as a downstream mediator of TORC1–S6K signaling. Repressing Syx13 in the fat body by double-stranded RNA interference (RNAi) induced the enlarged multilamellar lysosomes, while overexpressing Syx13 suppressed them. Further, suppression of TORC1–S6K–Syx13 signaling reduced age-associated chronic inflammation, the decline in ability to clear bacteria and extended lifespan via the endolysosomal control of regulatory PGRP-LC isoform. Moreover, repression of inflammation in mid-adulthood, by knocking down the *Drosophila* NF-κB like transcription factor Relish in the fat body, enhanced bacterial clearance and increased fly lifespan. Additionally, we found a notable sexual dimorphism in the fat body inflammaging and its response to reduced TORC1–S6K activity, which may contribute to the observed distinct lifespan effects of S6K in males and females. In mice, Rapa treatment increased expression of Syntaxin 12/13 (Stx12) in liver. Comparing the hepatic proteome and transcriptome of Rapa-treated mice with the transcriptome of S6K1-deficient mice showed alleviated immune processes in the aged liver as common health outcome of TORC1–S6K inhibition. Rapa treatment in mouse liver also decreased age-related activation of RelB and NF-κB2, core components of noncanonical NF-κB signaling. These findings define Stx12 as a downstream effector of TORC1–S6K signaling, highlighting a crucial role of the endolysosomal system in inflammaging, immunosenescence and longevity.

## Results

### S6K activity in the fat body is essential for longevity

**Fig. 1:**
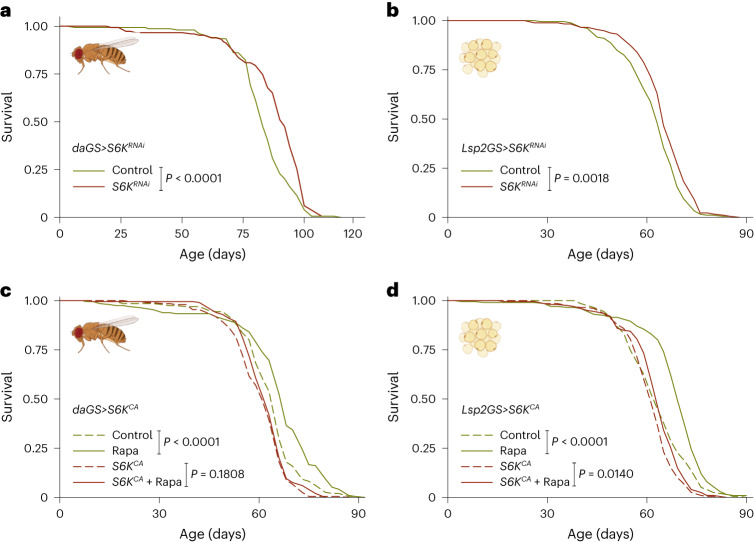
S6K activity in the fat body of adult flies determines longevity. **a**, Adult-onset repression of S6K ubiquitously using *daGS*>*S6K*^*RNAi*^ extended lifespan (*P* = 4.94 × 10^−6^, *n* = 150). **b**, Adult-onset repression of S6K in the fat body using *Lsp2GS*>*S6K*^*RNAi*^ extended lifespan (*n* = 180). **c**, Rapa extended lifespan of control flies, but not of flies with ubiquitous overexpression of constitutively active S6K (*daGS*>*S6K*^*CA*^). Ubiquitous overexpression of constitutively active S6K significantly attenuated the response to Rapa treatment (Rapa: *P* = 2.04 × 10^−5^, *daGS*>*S6K*^*CA*^ induction: *P* = 3.21 × 10^−5^, interaction *P* = 0.0236; *n* = 200). **d**, Adult-onset S6K activation in the fat body (*Lsp2GS*>*S6K*^*CA*^) significantly attenuated Rapa-related longevity (Rapa: *P* = 1.07 × 10^−7^, *Lsp2GS*>*S6K*^*CA*^ induction: *P* = 1.58 × 10^−5^, interaction *P* = 0.0331; *n* = 200). log-rank test and CPH test. Representative survival curve of two independent assays.

**Extended Data Fig. 1:**
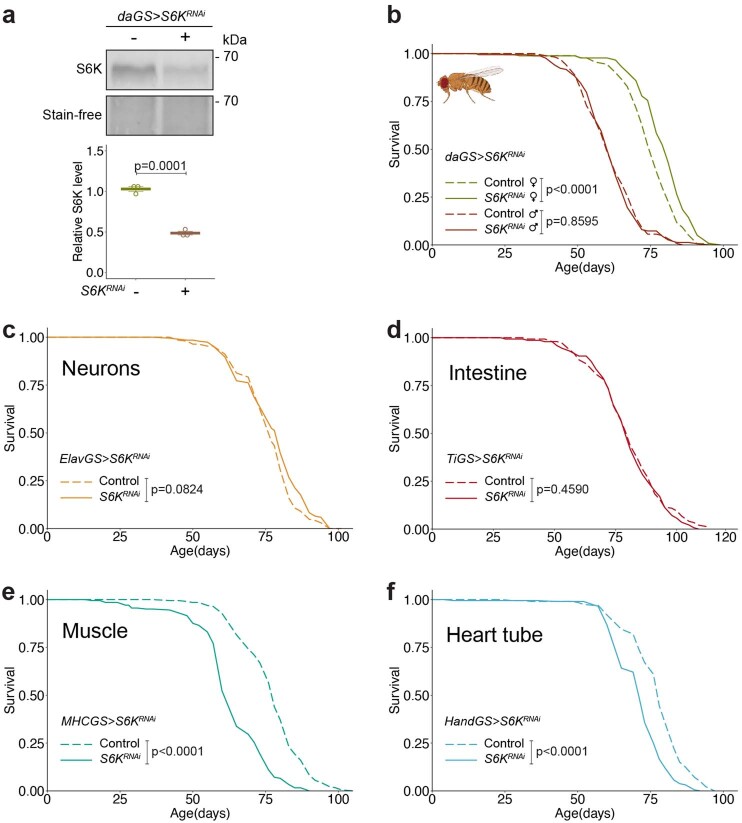
Downregulation of S6K activity ubiquitously in male flies or in the neurons, intestine, muscle, or heart tube of female flies does not extend lifespan. **a**, Adult-onset expression of S6K RNAi ubiquitously using *daGS* > *S6K*^*RNAi*^ repressed S6K level (n = 3). **b**, Adult-onset repression of S6K ubiquitously using *daGS* > *S6K*^*RNAi*^ extended lifespan in female flies, but not in males (gender: p < 2e-16, *Lsp2GS* > *S6K*^*RNAi*^ induction: p = 0.0001, interaction p = 0.0240; n = 180). **c-d**, Adult-onset repression of S6K in the neurons using *elavGS* > *S6K*^*RNAi*^ (**c**, n = 200) or in the intestine using *TiGS* > *S6K*^*RNAi*^ (**d**, n = 150) did not affect lifespan. **e-f**, Adult-onset repression of S6K in the muscle using *MHCGS* > *S6K*^*RNAi*^ (**e**, p = 3.936e-28, n = 200) or in the heart tube using *HandGS* > *S6K*^*RNAi*^ (**f**, p = 1.155e-12, n = 200) shortened lifespan. Each data point represents an average value per 20 fly heads **(a)**. Two-sided one-way ANOVA **(a)**. CPH analysis **(b)**. Log-rank test **(b-f)**. Representative survival curve of one independent assay **(b-f)**.

To address whether downregulation of S6K prolongs lifespan in *Drosophila*, we used the inducible GeneSwitch system in combination with an S6K RNAi line (Extended Data Fig. 1a) to knock down S6K expression specifically in adult female flies. Ubiquitous downregulation of S6K using *a daGS* driver resulted in a small but significant increase in lifespan (Fig. 1a and Extended Data Fig. 1b), demonstrating that, as in *Caenorhabditis* *elegans* and mice^16,28^, reduced S6K can extend lifespan in *Drosophila*. To identify the tissue(s) in which reduced S6K acted to extend lifespan, we employed tissue-specific GeneSwitch drivers to suppress S6K in fat body (*Lsp2GS*), neurons (*ElavGS*), intestine (*TiGS*), muscle (*MHCGS*) and heart tube (*HandGS*) of adult flies. Repression of S6K activity in neurons and intestine did not affect lifespan (Extended Data Fig. 1c,d), while in muscle and heart tube it shortened it (Extended Data Fig. 1e,f). Lifespan was extended only upon fat-body-specific repression of S6K (Fig. 1b), indicating that S6K in the fat body is limiting for lifespan. Lifespan extension by Rapa can be blocked by ubiquitous S6K activation^3^. We therefore tested whether activating S6K just in fat body tissue was sufficient to block the effect of Rapa on survival. Administration of Rapa to flies that overexpressed a constitutively active S6K protein (S6K^CA^) specifically in the fat body, similar to ubiquitous activation (Fig. 1c), was sufficient to mostly block Rapa-mediated lifespan extension (Fig. 1d). The fat body is therefore a key tissue regulating survival downstream of TORC1 and S6K.

### S6K activity in the fat body does not regulate gut health

**Extended Data Fig. 2:**
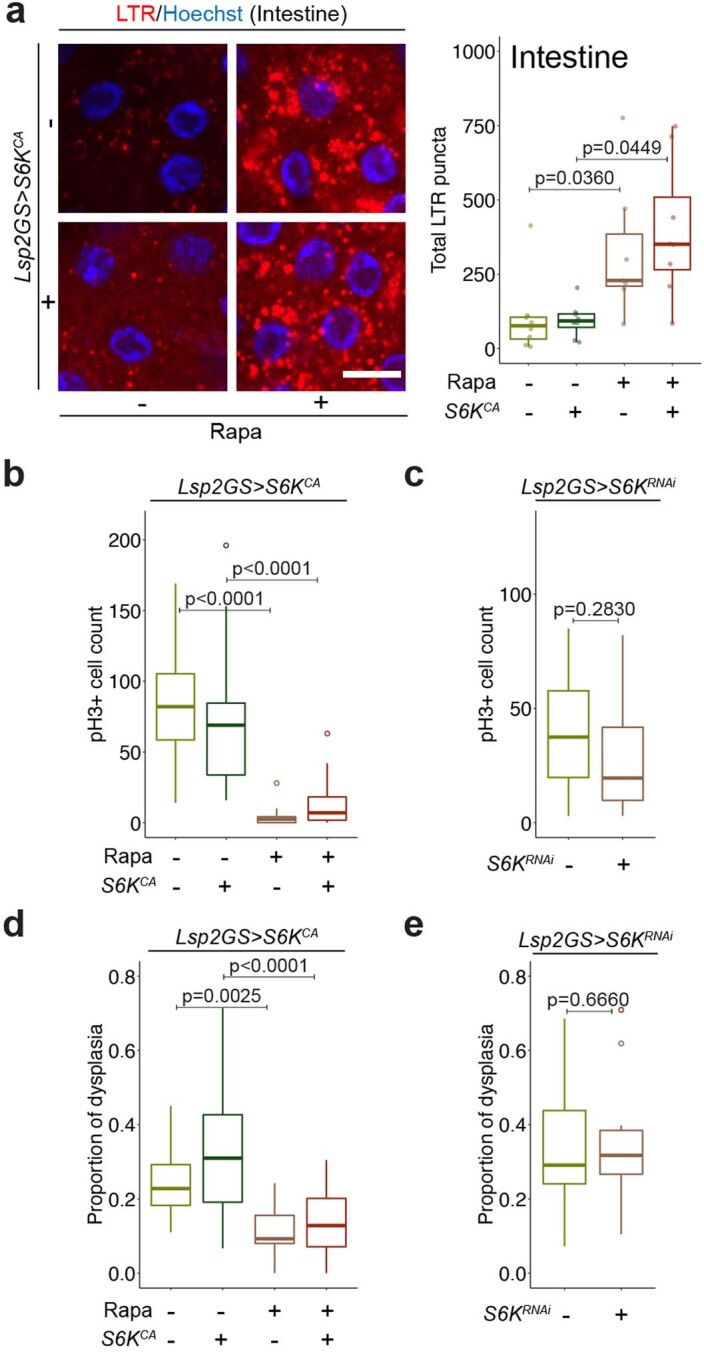
S6K activity in the fat body does not affect intestinal health. **a**, Rapamycin treatment induced acid organelles in the intestine of young (day 20) flies. Overexpression of constitutively active S6K specifically in the fat body (*Lsp2GS* > *S6K*^*CA*^) did not affect this phenotype, depicted by lysotracker staining (rapamycin: p = 1.338e-06, *Lsp2GS* > *S6K*^*CA*^ induction: p = 0.8463, interaction p = 0.9474; n = 8). **b**, Rapamycin treatment suppressed dividing intestinal stem cells of young (day 20) flies. Overexpression of constitutively active S6K specifically in the fat body (*Lsp2GS* > *S6K*^*CA*^) did not affect this phenotype, depicted by pH3 staining in the intestine (rapamycin: p = 2.9e-12, *Lsp2GS* > *S6K*^*CA*^ induction: p = 0.8310, interaction p = 0.1610; n = 21 in control group, 23 in *S6K*^*CA*^ group, 20 in Rapa group and Rapa+*S6K*^*CA*^ group). **c**, RNAi mediated downregulation of S6K activity specifically in the fat body (*Lsp2GS* > *S6K*^*RNAi*^) did not affect dividing intestinal stem cells of young (day 20) flies, depicted by pH3 staining in the intestine (n = 21 in control group and n = 23 in *S6K*^*RNAi*^ group). **d**, Rapamycin treatment suppressed intestinal dysplasia of old (day 50) flies. Overexpression of constitutive active S6K specifically in the fat body (*Lsp2GS* > *S6K*^*CA*^) did not affect this phenotype (rapamycin: p = 4.02e-08, *Lsp2GS* > *S6K*^*CA*^ induction: p = 0.1340, interaction p = 0.3960; n = 19 in control group, 18 in *S6K*^*CA*^ group, 17 in Rapa group and 20 in Rapa+*S6K*^*CA*^ group). **e**, RNAi mediated downregulation of S6K activity specifically in the fat body (*Lsp2GS* > *S6K*^*RNAi*^) did not affect intestinal dysplasia of old (day 50) flies (n = 15). For box plot, the centre is the median, the lower and upper bounds correspond to the first and third quartiles, the whiskers extend up to 1.5 times the interquartile range, and the minima and maxima are the observed minima and maxima. Each data point represents an average value per intestine. Two-sided linear mixed model followed by Tukey’s multiple comparison test. Scale bar, 10 μm.

The *Drosophila* intestine plays a crucial role in TOR-dependent longevity^11,29–32^. Treating flies with Rapa improved gut health by increasing intestinal autophagy, reducing intestinal stem cell (ISC) turnover, age-related gut dysplasia and leakiness^11,29–31^. Considering the crosstalk between the fat body and the gut^33,34^, we tested whether modifying S6K activity in the fat body affected intestinal health. As expected, Rapa increased the number of lysosome- and autophagy-associated LysoTracker-positive puncta^35^ in the gut, but activation of S6K in the fat body did not block this increase (Extended Data Fig. 2a), suggesting that S6K activity in the fat body does not affect intestinal autophagy. Next, we performed phospho-histone H3 (pH3) immunostaining of fly guts, as a proxy for proliferating ISCs^36^. Neither elevated S6K activity in combination with Rapa nor reduced S6K level in the fat body affected the number of proliferating ISCs when compared to the respective controls (Extended Data Fig. 2b,c). Consistent with previous findings^11,12^, Rapa treatment protected against age-related gut dysplasia, but enhancing S6K activity in the fat body did not block this effect, nor was it reduced by fat-body-specific S6K repression (Extended Data Fig. 2d,e). In summary, lowered S6K activity in the fat body did not increase longevity by improving gut health.

### Proteomics analysis of fat bodies with altered S6K activity

**Extended Data Fig. 3:**
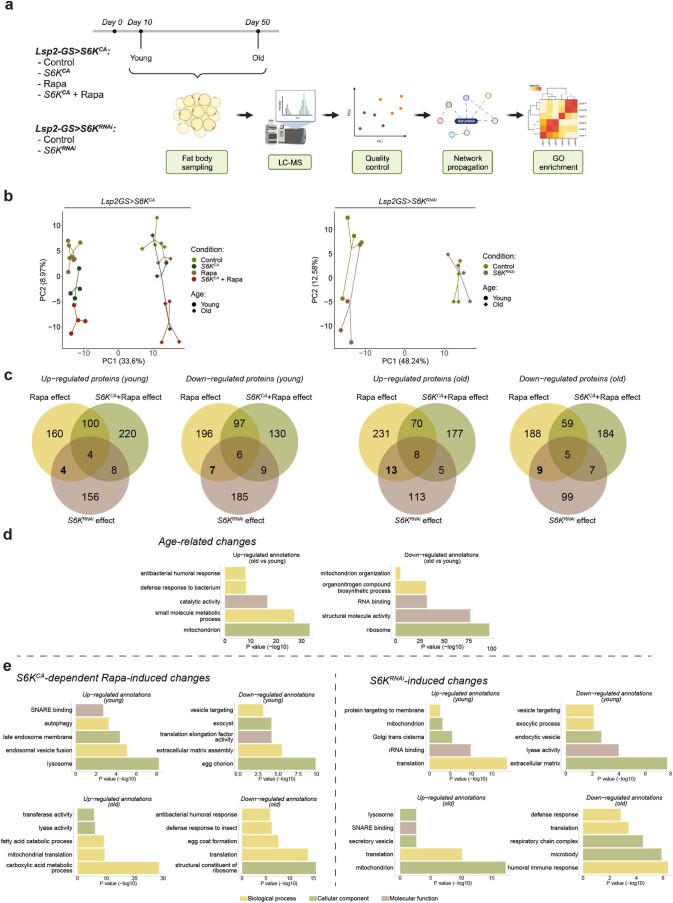
Proteomics analysis of TORC1-S6K dependent changes in the fly fat body. **a**, Outline of the proteomics experiment. **b**, Principal component analysis projections of proteomic replicates of *Lsp2GS* > *S6K*^*CA*^ (left) and *Lsp2GS* > *S6K*^*RNAi*^ (right), showing separation according to treatments and age. Each replicate represents a pool of five fat bodies**. c**, Venn diagram showing the number of up- and down-regulated proteins (p < 0.05) of each comparison at young and old age. ‘Rapa effect’ comprises differentially expressed proteins between Control *vs* Rapa; ‘*S6K*^*CA*^+Rapa effect’ comprises *S6K*^*CA*^ *vs S6K*^*CA*^+Rapa*;* and ‘*S6K*^*RNAi*^ effect’ comprises differentially expressed proteins between Control *vs S6K*^*RNAi*^. **d**, Representation of age-related changes on Gene Ontology (GO) terms between young and old age groups in the Control treatment group of *Lsp2GS* > *S6K*^*CA*^ dataset after network propagation. Colour indicates the categories of the terms. **e**, Representation of Gene Ontology (GO) terms in *Lsp2GS* > *S6K*^*CA*^ dataset (left) and *Lsp2GS* > *S6K*^*RNAi*^ dataset (right) after network propagation. S6K^CA^-dependent Rapa-induced annotations were selected if the p-value of Control *vs* Rapa annotation is at least 100 times greater than the p-value of *S6K*^*CA*^ *vs S6K*^*CA*^+Rapa annotation (EtOHvsRapa.log10_pvalue - RUvsRURapa.log10_pvalue > 2). Colour indicates the categories of the terms.

The *Drosophila* fat body is functionally equivalent to mammalian liver and white adipose tissue and is the central organ for metabolism and immune responses^37^. To gain further insight into the effects of TORC1–S6K signaling in the fat body, we used unbiased tandem mass tag (TMT)-based proteomic profiling of fat bodies from day 10 (young) and day 50 (old) flies with elevated S6K activity in fat bodies (*Lsp2GS*>*S6K*^*CA*^) in combination with Rapa treatment, or flies with repressed S6K level in fat bodies (*Lsp2GS*>*S6K*^*RNAi*^, Extended Data Fig. 3a). Principal component analysis revealed clear separation among age and treatment (Extended Data Fig. 3b). We detected a total of 4,101 proteins from the *Lsp2GS*>*S6K*^*CA*^ proteomics assay and 4,809 proteins from the *Lsp2GS*>*S6K*^*RNAi*^ proteomics assay (Supplementary Table 1). To explore S6K-dependent alterations in expression, we filtered the proteins that were significantly changed (*P* < 0.05) by both Rapa treatment and S6K manipulations, and these were associated with endocytosis, RNA binding processes and extracellular matrix in young fat bodies, and with mitochondria-, translation- and immune-related proteins in old fat bodies (Extended Data Fig. 3c and Supplementary Table 2).

To further enhance the resolution of our analysis, we conducted network propagation analysis^38^ to incorporate information on protein-protein interactions. We then identified functional categories and clustered them into age-related (Extended Data Fig. 3d), as well as Rapa-induced, S6K-dependent terms and S6K-inhibition-induced terms (Extended Data Fig. 3e). In old fat bodies, there was a marked upregulation of processes related to mitochondria, catalytic/metabolic activities and immune functions compared to young fat bodies. Conversely, there was a noticeable downregulation in ribosome-related processes, structural molecule activity and RNA binding (Extended Data Fig. 3d). Additionally, in young fat bodies, the inhibition of TORC1–S6K signaling led to an upregulation of processes associated with vesicle fusion, endosomes/lysosomes and translation, while processes related to oogenesis, the extracellular matrix and exocytosis were downregulated. In contrast, in the old fat bodies, inhibition of TORC1–S6K signaling resulted in an upregulation of terms related to translation, lysosomes and mitochondria, whereas immune functions, translation processes and respiratory chain complex activities were suppressed (Extended Data Fig. 3e).

### S6K does not affect fecundity, translation or lipid storage

**Extended Data Fig. 4:**
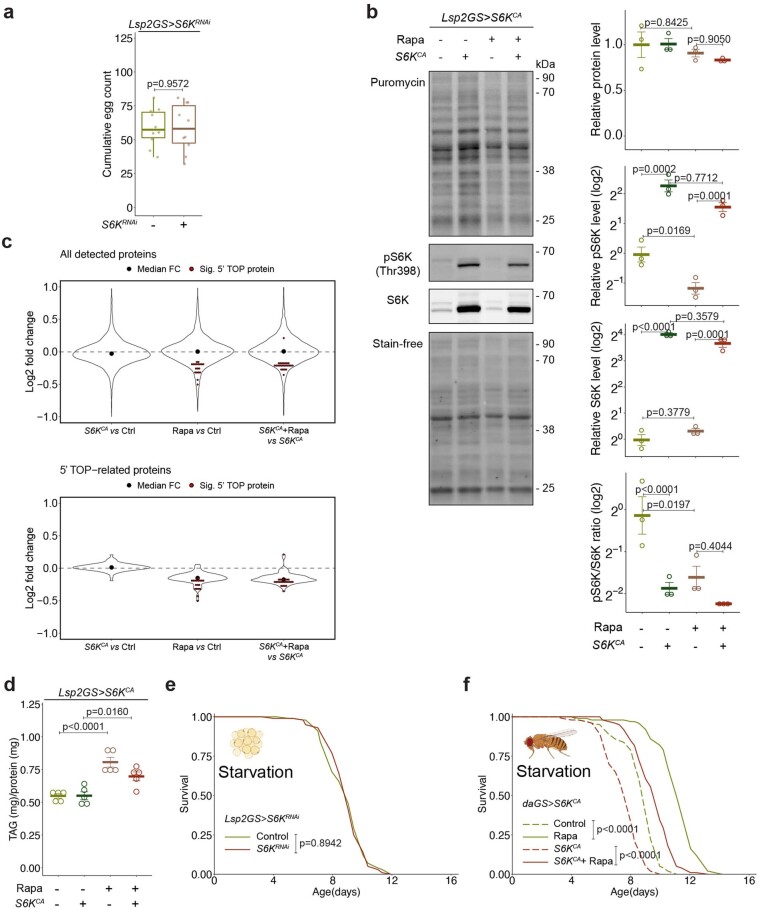
TORC1-S6K signalling in the fat body does not affect egg laying, global and 5’ TOP-related genes translation rate, TAG storage or starvation resistance. **a**, Repression of S6K in the fat body using *Lsp2GS* > *S6K*^*RNAi*^ did not affect cumulative egg laying (n = 10). **b**, Rapamycin treatment or overexpression of constitutively active S6K specifically in the fat body (*Lsp2GS* > *S6K*^*CA*^) did not affect the overall level of newly synthesized proteins, depicted by puromycin immunoblotting (rapamycin: p = 0.1307, *Lsp2GS* > *S6K*^*CA*^ induction: p = 0.6792, interaction p = 0.6168). Overexpression of S6K elevated both pS6K and total S6K level. Rapamycin treatment repressed pS6K level (rapamycin: p = 0.0018, *Lsp2GS* > *S6K*^*CA*^ induction: p = 1.551e-06, interaction p = 0.3253) and pS6K/S6K ratio (rapamycin: p = 0.0090, *Lsp2GS* > *S6K*^*CA*^ induction: p = 0.0023, interaction p = 0.0738) but did not affect total S6K level (rapamycin: p = 0.9751, *Lsp2GS* > *S6K*^*CA*^ induction: p = 4.343e-09, interaction p = 0.0398; n = 3). **c**, Rapamycin treatment or overexpression of S6K (*Lsp2GS* > *S6K*^*CA*^) did not affect the overall protein expression level in the fat body proteome of young flies (upper panel). Although rapamycin treatment repressed expression of genes containing 5’ TOP motif (‘Rapa *vs* Ctrl’, bottom panel), activation of specifically in the fat body (*Lsp2GS* > *S6K*^*CA*^) did not block the rapamycin effect (‘*S6K*^*CA*^+Rapa *vs S6K*^*CA*^’, bottom panel). **d**, Rapamycin increased triglyceride (TAG) storage of young flies. Expression of S6K in the fat body (*Lsp2GS* > *S6K*^*CA*^) did not block the effect of rapamycin on TAG storage (rapamycin: p = 5.36e-06, *Lsp2GS* > *S6K*^*CA*^ induction: p = 0.0957, interaction p = 0.0890; n = 5). **e**, Starvation resistance was not affected by downregulation of S6K in the fat body (*Lsp2GS* > *S6K*^*RNAi*^; n = 100). **f**, Rapamycin increased starvation resistance of young flies. Overexpression of S6K (*daGS* > *S6K*^*CA*^) did not block the effect of rapamycin on starvation resistance (rapamycin: p < 2e-16, *daGS* > *S6K*^*CA*^ induction: p = 8.78e-14, interaction p = 0.5770; n = 100). For box plot **(a)**, the centre is the median, the lower and upper bounds correspond to the first and third quartiles, the whiskers extend up to 1.5 times the interquartile range, and the minima and maxima are the observed minima and maxima. Data are displayed as mean ± s.e.m. **(b, d)**, or violin plot with median value **(c)**. Each data point represents an average value per vial **(a)**, per five fat bodies **(b)**, or per five whole flies **(d)**. Two-sided Student’s t- test **(a)**. Two-sided linear mixed model with **(b)** or without **(d)** log transformation followed by Tukey’s multiple comparison test. Log-rank test **(e-f)** and CPH analysis **(f)**.

The fat body is important for oogenesis and reproduction, and reduced reproduction is often associated with increased longevity^39^. As oogenesis was identified as a significantly enriched term in the proteomics analysis (Extended Data Fig. 3e), we measured egg laying of females with fat-body-specific S6K downregulation and found that fecundity was not affected (Extended Data Fig. 4a). Reduced reproduction is therefore unlikely to contribute to S6K-related lifespan extension, consistent with the findings for Rapa^3^.

S6K is a regulator of protein synthesis, and protein translation was identified as an enriched term in the proteomics analysis (Extended Data Fig. 3e). Furthermore, reduced protein synthesis has been associated with longevity in several invertebrate longevity models. To address whether S6K or Rapa affected global protein synthesis in the fly fat body, we performed puromycin incorporation assays. Although Rapa treatment repressed phosphorylation of S6K at Thr398 (pS6K) and activation of S6K elevated both pS6K and total S6K level, puromycin incorporation was not affected by Rapa treatment nor by S6K activation upon Rapa feeding (Extended Data Fig. 4b), suggesting that TORC1–S6K signaling does not change global translation rates in fat bodies. Consistently, deletion of S6K and chronic Rapa feeding also had no effect on global translation rates in mice^40,41^. TORC1 specifically controls translation of proteins that contain a 5′-terminal oligopyrimidine motif (5′TOP) in their messenger RNA^34^. Accordingly, although proteins with a 5′TOP motif in their mRNA were significantly downregulated in the fat body of Rapa treated animals, activation of S6K did not block this effect (Extended Data Fig. 4c), consistent with previous findings^42,43^.

Lipid metabolism has been implicated in the aging process^44^, and Rapa increases lipid storage in flies^3^. However, S6K activation did not alter Rapa-induced triglyceride accumulation in the fat body (Extended Data Fig. 4d). Moreover, repressing S6K in the fat body did not affect survival under starvation, and ubiquitous S6K activation did not attenuate Rapa-induced starvation resistance (Extended Data Fig. 4e,f). Thus, TORC1–S6K-dependent longevity cannot be explained by an effect on triglyceride accumulation.

### TORC1–S6K signaling affects lysosomal morphology

**Fig. 2:**
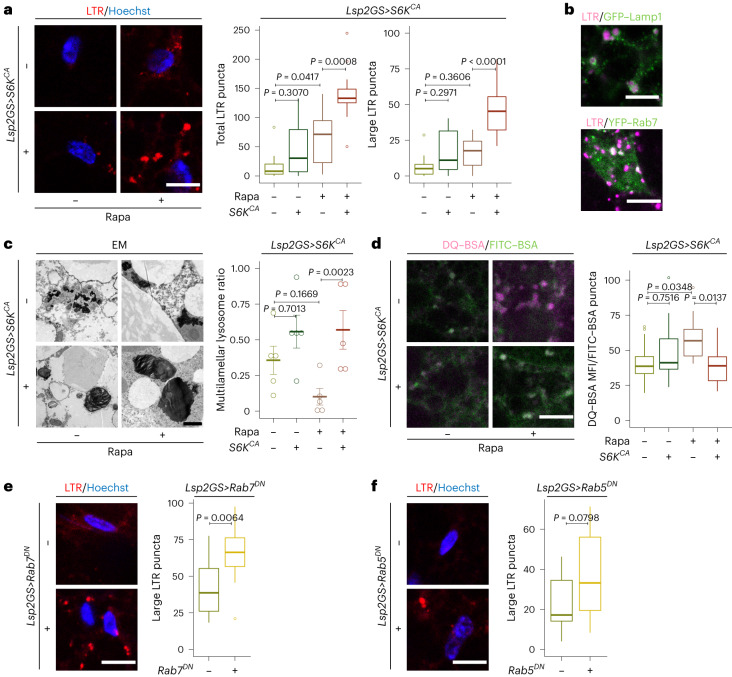
TORC1–S6K signaling affects lysosomal morphology and function in the fat body. **a**, LysoTracker staining of fat bodies from young (day 10) flies. Fat-body-specific, adult-onset overexpression of constitutively active S6K (*Lsp2GS*>*S6K*^*CA*^) significantly increased acidic organelle amount and size in response to Rapa treatment (total puncta: Rapa: *P* = 1.338 × 10^−6^, *Lsp2GS*>*S6K*^*CA*^ induction: *P* = 0.0001, interaction *P* = 0.0946; large puncta: Rapa: *P* = 2.896 × 10^−5^, *Lsp2GS*>*S6K*^*CA*^ induction: *P* = 1.618 × 10^−5^, interaction *P* = 0.0246; *n* = 12). **b**, LysoTracker-positive enlarged acidic organelles (magenta) colocalized with the lysosomal marker Lamp1 (*Lsp2GS*>*S6K*^*CA*^;*GFP–Lamp1*, green, top) and partially colocalized with Rab7 (*Lsp2GS*>*S6K*^*CA*^;*YFP–Rab7*, green, bottom), a marker for late endosomes, in young flies treated with Rapa and overexpressing S6K^CA^. **c**, Electron microscopy (EM) images of fat bodies of young flies treated with Rapa and overexpressing S6K^CA^. Overexpression of S6K^CA^ in the fat body attenuated the effect of Rapa on the ratio of multilamellar to total lysosomal area (Rapa: *P* = 0.0395, *Lsp2GS*>*S6K*^*CA*^ induction: *P* = 0.2798, interaction *P* = 0.0388; *n* = 5). **d**, Rapa increased lysosomal degradation capacity in the fat body of young flies, depicted by DQ–BSA/FITC–BSA pulse-chase assay. Lysosomes were labeled by FITC–BSA. Overexpression of S6K (*Lsp2GS*>*S6K*^*CA*^) blocked the effect of Rapa on lysosomal degradation capacity (Rapa: *P* = 0.3278, *Lsp2GS*>*S6K*^*CA*^ induction: *P* = 0.1382, interaction *P* = 0.0051; *n* = 14) in fat bodies of young flies. **e**, LysoTracker staining of young fat bodies overexpressing dominant negative Rab7 (*Lsp2GS>Rab7*^*DN*^). Fat-body-specific overexpression of Rab7^DN^ significantly increased acidic organelle size (*P* = 0.0064; *n* = 12). **f**, Fat-body-specific overexpression of dominant negative Rab5 (*Lsp2GS>Rab5*^*DN*^) also increased acidic organelle size (*P* = 0.0798; *n* = 12). For box plot (a and **d**–**f**), the center is the median, the lower and upper bounds correspond to the first and third quartiles, the whiskers extend up to 1.5 times the interquartile range, and the minima and maxima are the observed minima and maxima. Data are displayed as mean ± s.e.m. (**c**). Each data point represents an average value per fat body. Two-sided linear mixed model (**a** and **d**–**f**) or two-sided negative binomial generalized linear model (**c**) followed by Tukey’s multiple comparison test. Scale bar, 10 μm (**a**, **b** and **d**–**f**) or 1 μm (**c**).

**Extended Data Fig. 5:**
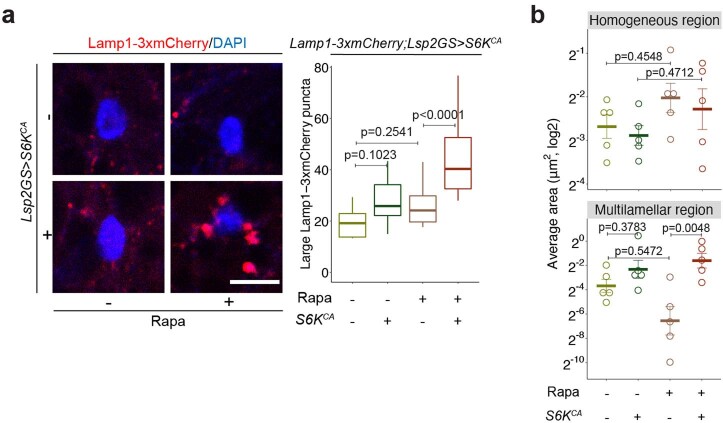
TORC1-S6K signalling affects lysosomal size and structure. **a**, Visualization of enlarged lysosomes in the fat body of young flies treated with rapamycin and overexpressing constitutively active S6K (*Lsp2GS* > *S6K*^*CA*^) using a *3xmCherry-Lamp1* reporter (rapamycin: p = 2.131e-05, *Lsp2GS* > *S6K*^*CA*^ induction: p = 2.042e-06, interaction p = 0.0468; n = 14). **b**, (Corresponding to Fig. 2c) Rapamycin treatment, but not overexpression of S6K, mildly increased the area of homogeneous, electron dense lysosomes, depicted by electron microscopy (rapamycin: p = 0.1410, *Lsp2GS* > *S6K*^*CA*^ induction: p = 0.9640, interaction p = 0.9640; upper panel). Rapamycin treatment mildly repressed the area of multilamellar lysosomes; however, overexpression of S6K attenuated the rapamycin effect on the multilamellar lysosomes (rapamycin: p = 0.1850, *Lsp2GS* > *S6K*^*CA*^ induction: p = 0.1090, interaction p = 0.1390; n = 5; bottom panel). For box plot **(a)**, the centre is the median, the lower and upper bounds correspond to the first and third quartiles, the whiskers extend up to 1.5 times the interquartile range, and the minima and maxima are the observed minima and maxima. Data are displayed as mean ± s.e.m. **(b)**. Each data point represents an average value per fat body. Two-sided linear mixed model **(a)** or two-sided negative binomial generalized linear model **(b)** followed by Tukey’s multiple comparison test. Scale bar, 10 μm.

Lysosome-related annotations were upregulated with the inhibition of TORC1–S6K activity in the young fat body (Extended Data Fig. 3e). Rapa treatment increased the number of LysoTracker-positive puncta in fat body cells (Fig. 2a, ‘Total LTR puncta’). Surprisingly, activation of S6K did not attenuate this increase, and instead led to a substantial accumulation and enlargement of acidic organelles (Fig. 2a). These S6K-dependent enlarged organelles were identified as lysosomes as indicated by *a GFP–Lamp1* reporter^45^, and late endosomes, indicated by *a YFP–Rab7* reporter (Fig. 2b). To measure changes in lysosomes directly, we used a *Lamp1–3xmCherry* reporter^46^, which marks lysosomes by an endogenous, promoter-driven, C-terminally mCherry-tagged Lamp1. mCherry-labeled lysosomes appeared as large puncta in fat bodies with S6K activation co-treated with Rapa (Extended Data Fig. 5a). Electron microscopy showed enlarged LysoTracker-positive puncta upon S6K activation as multilamellar lysosomes (Fig. 2c). However, large LysoTracker-positive puncta upon Rapa treatment were mainly due to the accumulation of more electron-dense lysosomes (Extended Data Fig. 5b). Thus, Rapa and S6K activation combined induce LysoTracker-positive puncta by two distinct mechanisms combined. Importantly, the ratio of multilamellar to total lysosomal area was ameliorated by Rapa treatment in an S6K-dependent manner (Fig. 2c). The multilamellar lysosomes suggests impaired lysosomal fusion or defective membrane function, which is typical of lysosomal storage diseases^47^. Thus, we next addressed lysosomal degradation capacity by performing a DQ–BSA (dye quenched-bovine serum albumin)/FITC–BSA (fluorescein isothiocyanate-bovine serum albumin) pulse-chase assay (Fig. 2d). Upon cleavage in the lysosome, DQ–BSA emits fluorescence. Rapa treatment resulted in an increased fluorescent signal, which was blocked by activation of S6K (Fig. 2d). Therefore, downregulation of TORC1 increased lysosomal degradation capacity in the fat body in an S6K-dependent manner.

**Extended Data Fig. 6:**
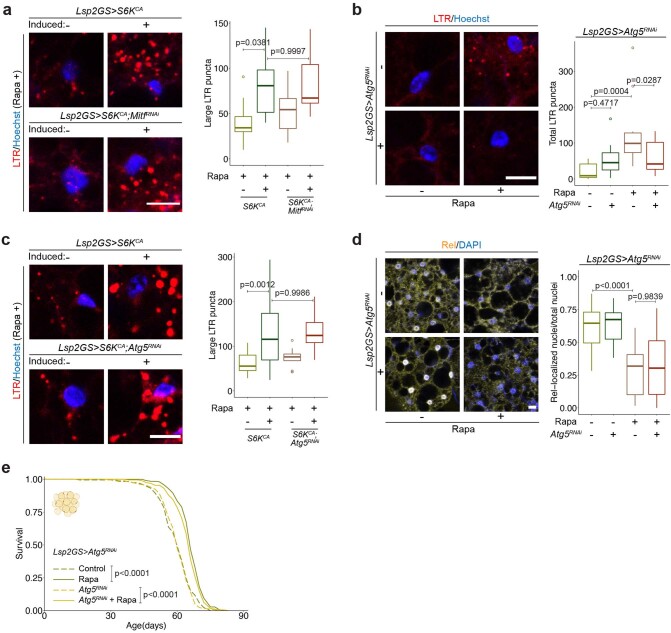
S6K-dependent lysosomal enlargement is not regulated by lysosomal biogenesis or autophagy in the fat body. **a**, RNAi mediated knock down of Mitf specifically in the fat body (*Lsp2GS* > *S6K*^*CA*^*;Mitf*^*RNAi*^) did not affect the enlarged lysosome phenotype of fat body tissue of S6K overexpression flies (*Lsp2GS* > *S6K*^*CA*^) treated with rapamycin, depicted by lysotracker staining (*Lsp2GS>Mitf*^*RNAi*^ induction: p = 0.4676, *Lsp2GS* > *S6K*^*CA*^ induction: p = 4.159e-05, interaction p = 0.5475; n = 12). **b**, Rapamycin treatment induced acid organelles in the fat body of young flies. RNAi mediated knock down of Atg5 specifically in the fat body (*Lsp2GS>Atg5*^*RNAi*^) blocked this phenotype, depicted by lysotracker staining (rapamycin: p = 0.0025, *Lsp2GS>Atg5*^*RNAi*^ induction: p = 0.2947, interaction p = 0.0035; n = 12). **c**, RNAi mediated knock down of Atg5 specifically in the fat body (*Lsp2GS* > *S6K*^*CA*^*;Atg5*^*RNAi*^) did not affect the enlarged lysosome phenotype of fat body tissue of S6K overexpression flies (*Lsp2GS* > *S6K*^*CA*^) treated with rapamycin, depicted by lysotracker staining (*Lsp2GS>Atg5*^*RNAi*^ induction: p = 0.6766, *Lsp2GS* > *S6K*^*CA*^ induction: p = 6.706e-06, interaction p = 0.5220; n = 12). **d**, RNAi mediated knock down of Atg5 specifically in the fat body (*Lsp2GS>Atg5*^*RNAi*^) did not affect Relish localisation in old fat bodies treated with rapamycin (rapamycin: p = 8.676e-10, *Lsp2GS>Atg5*^*RNAi*^ induction: p = 0.6189, interaction p = 0.9932; n = 14). **e**, RNAi mediated knock down of Atg5 specifically in the fat body (*Lsp2GS>Atg5*^*RNAi*^) did not block lifespan extension upon rapamycin treatment (rapamycin: p = 4.14e-15, *Lsp2GS>Atg5*^*RNAi*^ induction: p = 0.4130, interaction p = 0.4320; n = 200). For box plot, the centre is the median, the lower and upper bounds correspond to the first and third quartiles, the whiskers extend up to 1.5 times the interquartile range, and the minima and maxima are the observed minima and maxima. Each data point represents an average value per fat body. Two-sided linear mixed model followed by Tukey’s multiple comparison test **(a-d)**. Log-rank test and CPH analysis **(e)**. Representative survival curve of one independent assay **(e)**. Scale bar, 10 μm.

Lysosome biogenesis is a crucial mechanisms for regulating lysosome number and size^48^. Transcription factor EB (TFEB, termed Mitf in *Drosophila*) is a master regulator of lysosomal biogenesis. To test whether Mitf plays a role in S6K-dependent lysosomal enlargement we downregulated Mitf in fat bodies, but this did not block the formation of large LysoTracker-positive punctae in S6K^CA^-expressing flies treated with Rapa (Extended Data Fig. 6a). The lysosome is the terminal compartment for autophagy and endocytosis^48^. As autophagy plays a vital role during aging^9^, we investigated if defective autophagy caused the TORC1–S6K-dependent lysosomal changes. Consistent with previous findings^3^, repressing autophagy induction by *Lsp2GS>Atg5*^*RNAi*^ blocked Rapa-induced accumulation of LysoTracker-positive organelles in the fat body (Extended Data Fig. 6b), indicative of perturbed autophagy. However, the number of enlarged LysoTracker-positive organelles induced by S6K activation with Rapa treatment was not changed upon inhibition of Atg5 (Extended Data Fig. 6c), suggesting that the S6K-dependent lysosomal enlargement was not caused by autophagy dysfunction.

TORC1–S6K-dependent enlarged LysoTracker-positive organelles were partially colocalized with late endosomes (Fig. 2b). Thus, we next investigated if interfering with the endocytosis pathway would cause lysosomal enlargement. We impaired late endosome formation by expressing dominant-negative (DN) Rab7 (*Lsp2GS>Rab7*^*DN*^), which led to enlarged LysoTracker-positive organelles (Fig. 2e). Moreover, perturbation of early endosomes by DN Rab5 (*Lsp2GS>Rab5*^*DN*^) also mildly induced LysoTracker-positive organelle enlargement (Fig. 2f). In summary, interfering with endosomes but not autophagy caused a similar lysosomal phenotype as activation of S6K, suggesting that TORC1–S6K regulates lysosomal morphology via modulation of the endolysosomal system.

### S6K controls lysosomal structure through Syx13

**Fig. 3:**
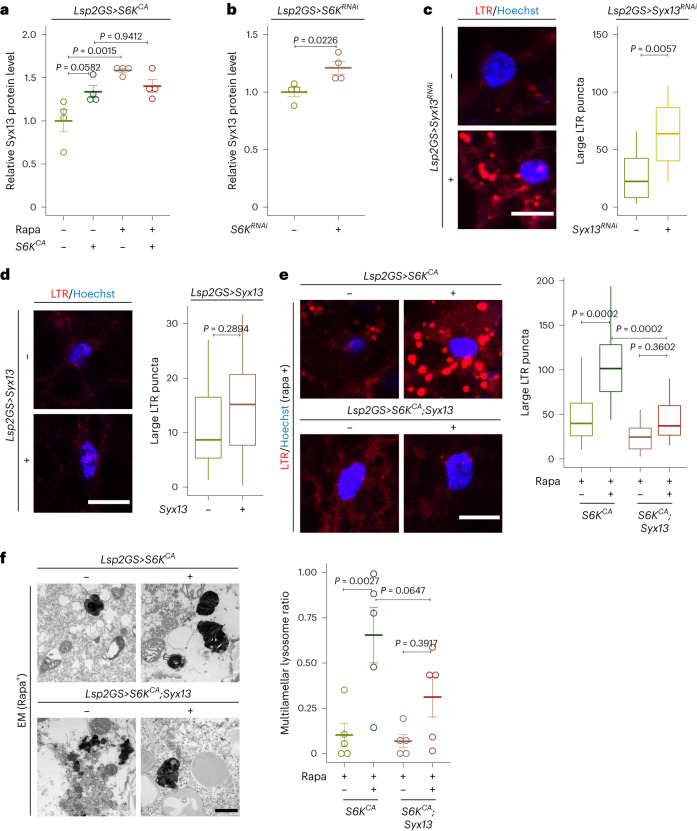
Syx13 is a downstream effector of TORC1–S6K signaling that regulates lysosomal structure in the fly fat body. **a**, Syx13 protein level was increased upon Rapa treatment in young fat body cells, and this increase was partly reverted by S6K overexpression (*Lsp2GS*>*S6K*^*CA*^*;* Rapa: *P* = 0.0003, *Lsp2GS*>*S6K*^*CA*^ induction: *P* = 0.0138, interaction *P* = 0.0086; *n* = 5). **b**, Syx13 protein level was increased upon S6K repression (*Lsp2GS*>*S6K*^*RNAi*^*;* *P* = 0.0226; *n* = 5) in young fat body cells. Syx13 protein levels were measured by mass spectrometry-based proteomics. **c**, Knockdown of Syx13 expression (*Lsp2GS>Syx13*^*RNAi*^) resulted in enlarged lysosomes in the fat body of young flies, depicted by LysoTracker staining (*P* = 0.0057; *n* = 14). **d**, Overexpression of Syx13 (*Lsp2GS>Syx13*) did not affect lysosomal enlargement (*P* = 0.2894; *n* = 12) in young fat bodies. **e**, Overexpression of Syx13 (*Lsp2GS*>*S6K*^*CA*^*;Syx13*) rescued the enlarged lysosomes of flies overexpressing S6K (*Lsp2GS*>*S6K*^*CA*^) treated with Rapa (*Lsp2GS*>*S6K*^*CA*^ induction: *P* = 5.559 × 10^−5^, *Lsp2GS>Syx13* induction: *P* = 6.083 × 10^−5^, interaction *P* = 0.0429; *n* = 12). **f**, Overexpression of Syx13 (*Lsp2GS*>*S6K*^*CA*^*;Syx13*) partially rescued the multilamellar lysosomes of S6K overexpressing (*Lsp2GS*>*S6K*^*CA*^) flies treated with Rapa, depicted by electron microscopy (*Lsp2GS*>*S6K*^*CA*^ induction: *P* = 0.0005, *Lsp2GS>Syx13* induction: *P* = 0.7989, interaction *P* = 0.3068; *n* = 5). Data are displayed as mean ± s.e.m. (**a**, **b** and **f**). For box plot (**c**–**e**), the center is the median, the lower and upper bounds correspond to the first and third quartiles, the whiskers extend up to 1.5 times the interquartile range, and the minima and maxima are the observed minima and maxima. Each data point represents an average value per five fat bodies (**a** and **b**) or per fat body (**c**–**f**). Two-sided linear mixed model (**a**–**e**) or two-sided negative binomial generalized linear model (**f**) followed by Tukey’s multiple comparison test. Scale bar, 10 μm (**c**–**e**) or 1 μm (**f**).

**Extended Data Fig. 7:**
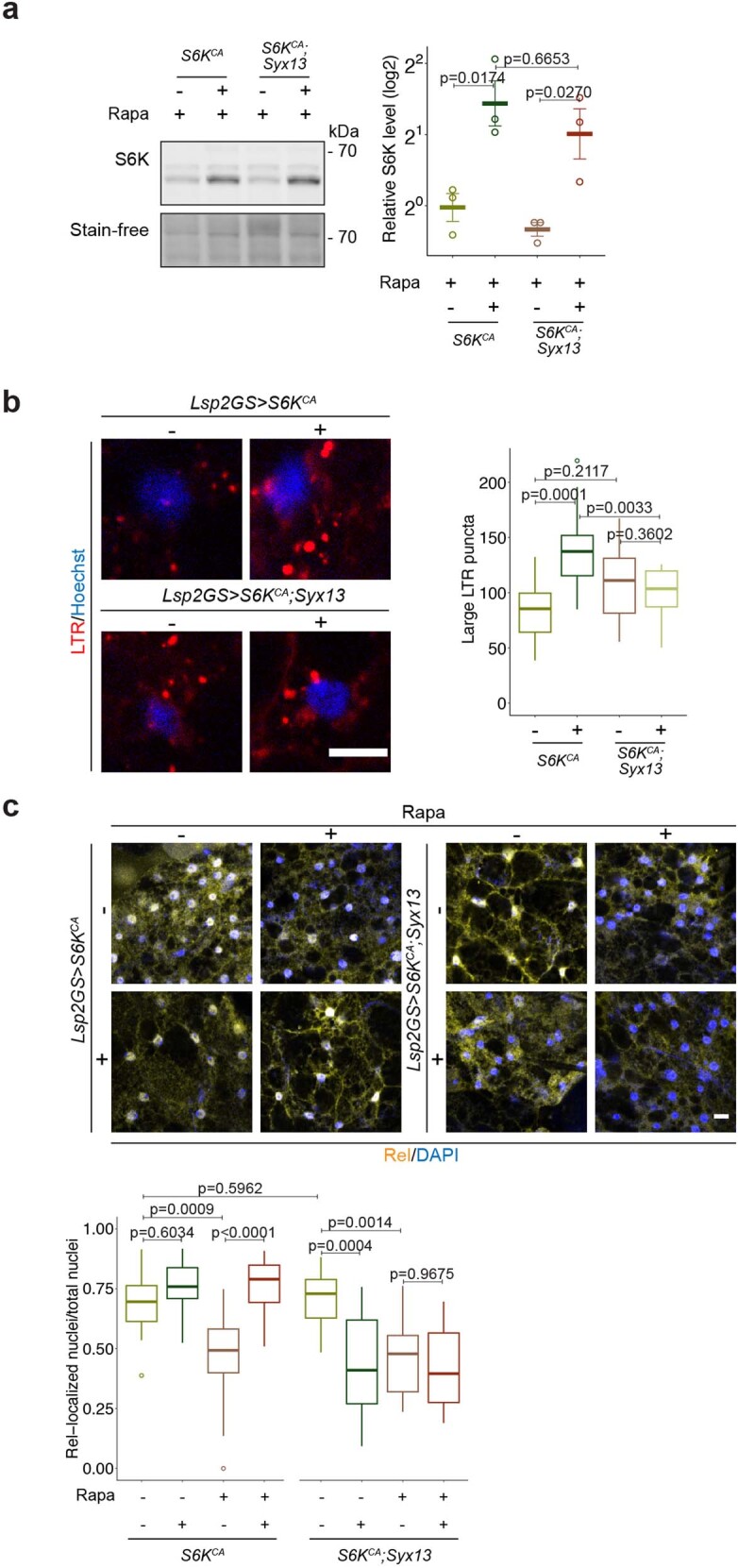
Syx13 regulates lysosomal morphology and inflammageing in the fly fat body. **a**, Overexpression of constitutively active S6K (*Lsp2GS* > *S6K*^*CA*^) increased total S6K level. Co-overexpression of Syx13 with S6K did not affect the effect of S6K overexpression on the total S6K level in fly fat body (*Lsp2GS* > *S6K*^*CA*^ induction: p = 0.0007, *Lsp2GS>Syx13* induction: p = 0.1955, interaction p = 0.8240; n = 3). **b**, Overexpression of constitutively active S6K (*Lsp2GS* > *S6K*^*CA*^) induced enlarged lysosomes in the fat body of young flies. Overexpression of Syx13 (*Lsp2GS* > *S6K*^*CA*^*;Syx13*) rescued the enlarged lysosomes of flies over-expressing S6K (*Lsp2GS* > *S6K*^*CA*^) (*Lsp2GS* > *S6K*^*CA*^ induction: p = 0.0046, *Lsp2GS>Syx13* induction: p = 0.2417, interaction p = 0.0002; n = 14). **c**, Overexpression of constitutive active S6K specifically in the fat body (*Lsp2GS* > *S6K*^*CA*^) blocked the effect of rapamycin on the age-related nuclear localisation of Relish (rapamycin: p = 0.0065, *Lsp2GS* > *S6K*^*CA*^ induction: p = 2.075e-05, interaction p = 0.0052; n = 14). Co- overexpression of Syx13 and S6K (*Lsp2GS* > *S6K*^*CA*^*;Syx13*) repressed the age-related nuclear localisation of Relish. Rapamycin treatment did not further suppress the effect of Syx13 and S6K co-overexpression on the age-related nuclear localisation of Relish (rapamycin: p = 0.0061, *Lsp2GS* > *S6K*^*CA*^*;Syx13* induction: p = 0.0014, interaction p = 0.0090; n = 14). Data are displayed as mean ± s.e.m. **(a)**. For box plot **(b-c)**, the centre is the median, the lower and upper bounds correspond to the first and third quartiles, the whiskers extend up to 1.5 times the interquartile range, and the minima and maxima are the observed minima and maxima. Each data point represents an average value per five fat bodies **(a)** or per fat body **(b-c)**. Two-sided two-way ANOVA with log transformation followed by Tukey’s multiple comparison test **(a)**. Two-sided linear mixed model followed by Tukey’s multiple comparison test for comparison of all conditions **(b)** or within each genotype separately **(c)**. Two-sided linear mixed model for comparison between *S6K*^*CA*^ and *S6K*^*CA*^*;Syx13* control groups **(c)**. Scale bar, 10 μm.

Proteins associated with vesicle fusion and vesicle membrane-related processes were regulated by TORC1–S6K signaling in the proteomics analysis (Extended Data Fig. 3e). Among them, protein levels of the SNARE protein Syx13 were upregulated both upon S6K knockdown and by Rapa treatment (Fig. 3a,b and Supplementary Table 2). Furthermore, the increase in Syx13 protein levels upon Rapa feeding was blocked upon activation of S6K (Fig. 3a), suggesting Syx13 as a potential downstream effector of TORC1–S6K in regulating lysosomal morphology. Consistent with this hypothesis, repression of Syx13 in young fat bodies caused lysosomal enlargement (Fig. 3c). In contrast, overexpression of Syx13 did not alter lysosomal morphology under basal conditions (Fig. 3d). To determine whether Syx13 was causally associated with the S6K-induced changes in lysosomal morphology, we increased both Syx13 expression and S6K activity in the fat body of flies that were treated with Rapa. Co-overexpression of Syx13 did not affect the efficiency of S6K overexpression (Extended Data Fig. 7a), but S6K-induced enlarged lysosomes (Fig. 3e and Extended Data Fig. 7b) and the associated multilamellar structure (Fig. 3f) were both diminished by Syx13 overexpression. Therefore, TORC1–S6K–Syx13 signaling mediated the lysosomal structural changes.

### S6K regulates inflammaging and immunosenescence

**Fig. 4:**
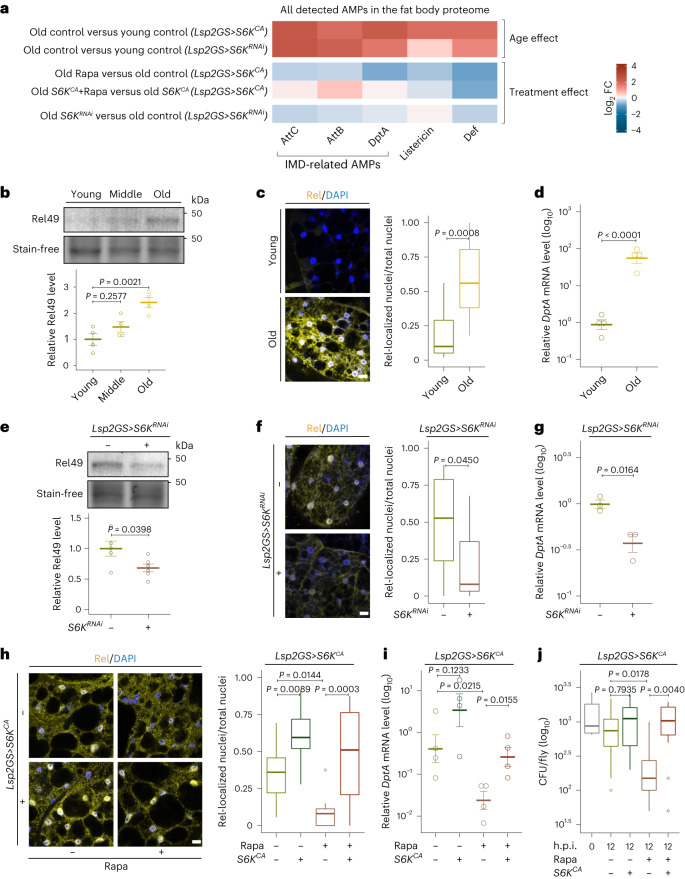
Immune aging is modulated by TORC1–S6K signaling in the fat body. **a**, log_2_ fold change (FC) across age and treatments of all AMPs detected in the fat body proteome. All AMPs accumulated with age and were repressed by Rapa treatment; S6K activation specifically blocked the effects of Rapa AMPs (*AttC*, *AttB* and *DptA)* downstream of the IMD pathway. AMPs associated with the IMD pathway were also repressed by S6K inhibition. **b**, Cleaved Relish (Rel49, 49 kDa) in fat bodies of young (day 10), middle (day 30) and old (day 50) flies (age effect *P* = 0.0034; *n* = 4). **c**,**d**, Relish (Rel) protein localization (**c**, *n* = 9 in young and *n* = 13 in old) and *DptA* transcript expression (**d**, *n* = 4) in fat bodies of young and old flies. **e**–**g**, S6K inhibition (*Lsp2GS*>*S6K*^*RNAi*^) suppressed the age-related increase in activated Rel49 (**e**, *n* = 5), accumulation of Relish in the nucleus (**f**, *n* = 14), and the increase in *DptA* expression (**g**, *n* = 3) in fat bodies of old (day 50) flies. **h**,**i**, Rapa treatment suppressed age-related Relish localization (**h**) and the increase in *DptA* (**i**). Overexpression of S6K (*Lsp2GS*>*S6K*^*CA*^) blocked the effect of Rapa on Relish localization (**h**, Rapa: *P* = 0.0025, *Lsp2GS*>*S6K*^*CA*^ induction: *P* = 1.339 × 10^−6^, interaction *P* = 0.2345; *n* = 14) and *DptA* expression (**i**, *n* = 4) in fat bodies of old (day 50) flies. **j**, Rapa treatment improved bacterial clearance in old (day 50) flies infected with *Ecc15*, indicated by colony forming unit (CFU) assay. This effect was blocked by S6K overexpression (*Lsp2GS*>*S6K*^*CA*^) (Rapa: *P* = 0.0185, *Lsp2GS*>*S6K*^*CA*^ induction: *P* = 0.0024, interaction *P* = 0.0620; *n* = 12). Data are displayed as mean ± s.e.m. (**b**, **d**, **e**, **g** and **i**). For box plot (**c**, **f**, **h** and **j**), the center is the median, the lower and upper bounds correspond to the first and third quartiles, the whiskers extend up to 1.5 times the interquartile range, and the minima and maxima are the observed minima and maxima. Each data point represents an average value per fat body (**c**, **f** and **h**), per five fat bodies (**b**, **d**, **e**, **g** and **i**) or per three whole flies (**i**). Two-sided one-way analysis of variance (ANOVA) followed by Dunnett’s multiple comparison test (**b**). Two-sided linear mixed model (**c**, **f** and **h**) or two-sided two-way ANOVA with log transformation (**j**) followed by Tukey’s multiple comparison test. Two-sided Student’s *t*-test with (**d**, **g** and **i**) or without log-transformation (**e**). Scale bar, 10 μm.

Lysosomal function plays a crucial role in inflammatory and autoimmune disorders^49^. A growing body of evidence has established that aging can lead to a low-grade inflammation that is associated with increased mortality^22,50,51^. The fat body is a major immune organ of *Drosophila*, and the proteins upregulated in old fat bodies that were altered by TORC1–S6K signaling showed significant enrichment of immune-related Gene Ontology (GO) annotations (Extended Data Fig. 3d,e). Antimicrobial peptides (AMPs) are key defense molecules of the *Drosophila* innate immune system, and their levels in fat body increased with age and were reduced upon Rapa treatment in old animals (Fig. 4a). Activation of S6K abolished the Rapa effect on attacins (AttC and AttB) and diptericin (DptA), which are primarily controlled by the immune deficiency (IMD) pathway. These AMPs were also reduced by S6K inhibition in old fat bodies, suggesting that the activity of the IMD pathway might be influenced by TORC1–S6K signaling during aging.

In *Drosophila*, the IMD pathway is the primary immune response pathway. When activated, the NF-κB-like transcription factor Relish is cleaved in the cytoplasm, and the activated fragment of Relish translocates to the nucleus and induces the transcription of AMPs, such as *DptA* (refs. ^33,52,53^). In line with previous reports^33,54,55^, aged fat bodies exhibited elevated Relish cleavage (Fig. 4b), Relish-positive nuclei (Fig. 4c) and *DptA* expression (Fig. 4a,d), indicating that IMD signaling was activated during aging. These changes were significantly ameliorated by reduced S6K activity (Fig. 4e–g). Furthermore, Rapa treatment greatly suppressed the age-associated changes in Relish localization and *DptA* expression in fat bodies, while activation of S6K blocked these effects (Fig. 4h,i). Immunosenescence also occurs during aging, with a declining ability of old flies to clear pathogens^56,57^. We therefore investigated whether S6K plays a role in bacterial clearance upon systemic infection with *Ecc15*, a Gram-negative bacterium widely used to study *Drosophila* immune responses^58^. Rapa treatment increased bacterial clearance in old flies, and expression of S6K^CA^ specifically in fat bodies blocked this effect (Fig. 4j). These results suggest that TORC1–S6K signaling regulates age-associated activation of the IMD pathway and the decline of pathogen clearance.

**Extended Data Fig. 8:**
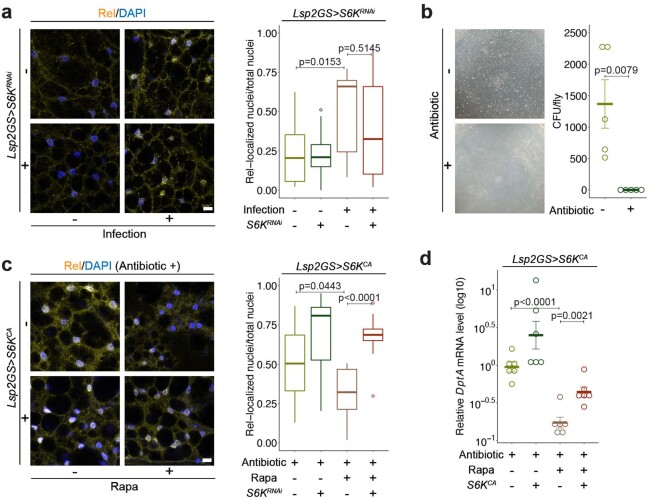
Reduced inflammageing with lowered TORC1-S6K activity is independent of bacterial load. **a**, Infection with *Ecc15* induced nuclear accumulation of Relish in young fat body cells. RNAi-mediated knockdown of S6K (*Lsp2GS* > *S6K*^*RNAi*^) did not affect the infection-induced localisation of Relish to the nucleus (infection: p = 0.0013, *Lsp2GS* > *S6K*^*RNAi*^ induction: p = 0.3499, interaction p = 0.3151; n = 12). **b**, Colony forming unit (CFU) assay confirmed effective bacterial growth repression by the antibiotic treatment with tetracycline and ampicillin (n = 5). **c**, Rapamycin treatment suppressed accumulation of Relish in the nucleus in the fat body of old flies treated with antibiotic. Overexpression of constitutively active S6K (*Lsp2GS* > *S6K*^*CA*^) blocked the effect of rapamycin on Relish localisation (rapamycin: p = 0.0264, *Lsp2GS* > *S6K*^*CA*^ induction: p = 7.826e-07, interaction p = 0.1300; n = 14). **d**, Rapamycin treatment suppressed *DptA* expression in old fat body cells. Overexpression of constitutively active S6K (*Lsp2GS* > *S6K*^*CA*^) induced *DptA* expression of flies treated with rapamycin and antibiotics (n = 6). For box plot **(a, c)**, the centre is the median, the lower and upper bounds correspond to the first and third quartiles, the whiskers extend up to 1.5 times the interquartile range, and the minima and maxima are the observed minima and maxima. Data are displayed as mean ± s.e.m. **(b, d)**. Each data point represents an average value per fat body **(a, c)**, per whole fly **(b)**, or per five fat bodies **(d**). Two-sided linear mixed model followed by Tukey’s multiple comparison test **(a, c)**. Mann-Whitney test **(b)**. Two-sided Student’s t-test with log transformation **(d)**. Scale bar, 10 μm.

Potential mechanisms of age-associated immune activation include increased intestinal permeability, chronic infection and ‘sterile inflammation’ caused by internal factors^22,59^. We next investigated whether S6K directly regulates IMD activation upon microbial infection. We therefore challenged young fat bodies ex vivo with *Ecc15*. Although *Ecc15* infection led to a clear induction in the number of Relish-positive nuclei under basal conditions, knockdown of S6K failed to repress this induction (Extended Data Fig. 8a), suggesting that the effect of S6K on the IMD pathway is indirect and dependent on age-dependent changes in fat bodies. We next explored whether activation of the IMD pathway by TORC1–S6K signaling is dependent on age-associated accumulation of bacteria^60^. Therefore, flies were treated with antibiotics from young age to eliminate bacterial accumulation during aging. Colony unit forming assay indicated that the antibiotic treatment dramatically reduced bacterial growth (Extended Data Fig. 8b). However, Rapa treatment still repressed the number of Relish-positive nuclei and *DptA* expression in an S6K-dependent manner (Extended Data Fig. 8c,d). These results show that TORC1–S6K signaling regulates age-associated activation of the IMD pathway independent of bacterial load.

### Endolysosomes mediate the Rapa effects on inflammaging

**Fig. 5:**
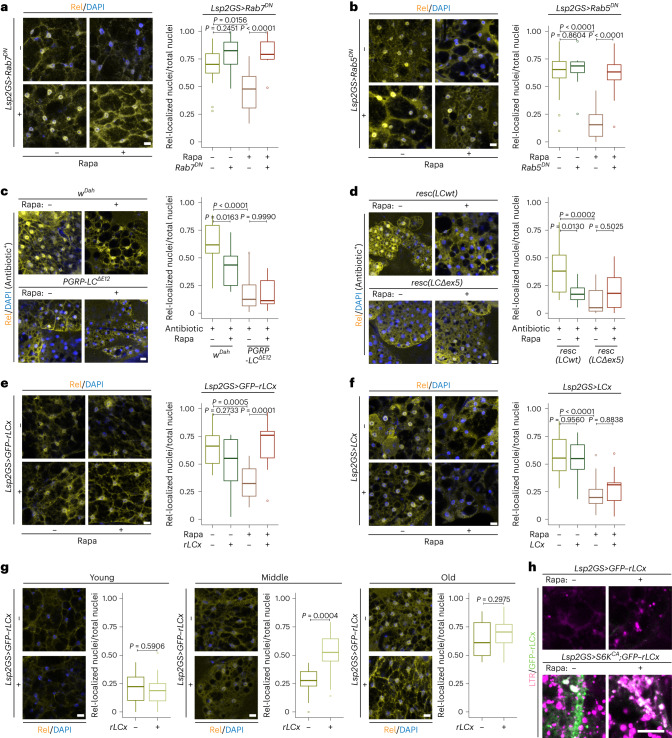
Reduced TORC1–S6K attenuates inflammaging via the endolysosomal regulation of rPGRP-LC in the fat body. **a**,**b**, Rapa treatment suppressed the age-related nuclear localization of Relish (Rel) in old fat body cells. Dominant-negative Rab7 (**a**, Rapa: *P* = 0.0638, *Lsp2GS>Rab7*^*DN*^ induction: *P* = 4.149 × 10^−6^, interaction *P* = 0.0160; *n* = 14) and Rab5 (**b**, Rapa: *P* = 4.467 × 10^−6^, *Lsp2GS>Rab5*^*DN*^ induction: *P* = 7.276 × 10^−6^, interaction *P* = 0.0003; *n* = 14) blocked the effect of Rapa on the age-related nuclear localization of Relish. **c**,**d**, Removing the full-length PGRP-LC (*PGRP-LC*^*ΔE12*^) and only the regulatory PGRP-LC isoforms by deleting exon 5 (*resc(LCΔex5)*) repressed the age-related nuclear localization of Relish and Rapa effect in the fat body of old flies treated with antibiotic (**c**, Rapa: *P* = 0.0355, *PGRP-LC*^*ΔE12*^: *P* = 7.925 × 10^−9^, interaction *P* = 0.0569; *n* = 9 in *PGRP-LC*^*ΔE12*^ group and *n* = 14 in other groups; **d**, Rapa: *P* = 0.1642, *resc(LCΔex5)*: *P* = 0.0028, interaction *P* = 0.0018; *n* = 14). **e**, Overexpression of regulatory PGRP-LC isoform (*Lsp2GS*>*GFP–rLCx*) suppressed the Rapa effect on Relish localization in old fat body cells (Rapa: *P* = 0.1921, *Lsp2GS*>*GFP–rLCx* induction: *P* = 0.0432, interaction *P* = 2.285 × 10^−5^; *n* = 14). **f**, Overexpression of canonical PGRP-LC isoform (*Lsp2GS*>*LCx*), did not affect the Rapa effect on Relish localization in old fat body cells (Rapa: *P* = 1.419 × 10^−8^, *Lsp2GS*>*LCx* induction: *P* = 0.8768, interaction *P* = 0.3832; *n* = 14). **g**, Overexpression of regulatory PGRP-LC isoform (*Lsp2GS*>*GFP–rLCx*) did not affect the nuclear localization of Relish in young (left, *n* = 14) and old (right, *n* = 14) fat body cells, but induced the nuclear localization of Relish in middle-aged (middle, *n* = 14) fat body cells. Time course experiments were performed separately using the same batch of flies. **h**, Regulatory PGRP-LC (green) colocalized with LysoTracker-positive enlarged acidic organelles (magenta) in the fat body of young flies treated with Rapa and overexpressing S6K^CA^ (*Lsp2GS*>*S6K*^*CA*^;*GFP–rLCx*, *n* = 14). For box plot, the center is the median, the lower and upper bounds correspond to the first and third quartiles, the whiskers extend up to 1.5 times the interquartile range, and the minima and maxima are the observed minima and maxima. Each data point represents an average value per fat body. Two-sided linear mixed model followed by Tukey’s multiple comparison test. Scale bar, 10 μm.

Given that TORC1–S6K–Syx13 signaling regulated lysosomal morphology, we hypothesized that age-related IMD activation in fat bodies could be an outcome of endolysosomal dysfunction. We therefore investigated if the endocytosis pathway contributes to TORC1–S6K-related regulation of Relish. Overexpression of DN Rab7 blocked the Rapa-induced decrease in the number of Relish-positive nuclei in the aged fat body (Fig. 5a). Moreover, impairment of early endosomes by expression of DN Rab5 attenuated TORC1-related Relish repression (Fig. 5b), suggesting that the endolysosomal system acts downstream of TORC1–S6K in regulating IMD activity.

Disrupting endosomal trafficking induces the accumulation of cytokine receptors and subsequently activates the IMD/NF-κB pathway in fly fat bodies^61^ and in human cells^62^. Peptidoglycan recognition protein LC (PGRP-LC) is a key upstream receptor of the IMD pathway in flies^53^. Loss of PGRP-LC strongly reduced the number of Relish-positive nuclei and there was no additional effect of Rapa treatment (Fig. 5c), suggesting a vital role of PGRP-LC in inflammaging. The *PGRP-LC* gene is a complex locus that produces both activating isoforms (PGRP-LC; LC, including LCx, LCy and LCa) and regulatory isoforms (rPGRP-LC; rLC, including rLCx, rLCy and rLCa)^61^. To address which isoform underlies the observed phenotype, we used a mutation that specifically deleted rPGRP-LC. Interestingly, lack of just the regulatory isoform was sufficient to block age-related accumulation of Relish-positive nuclei (Fig. 5d), similar to the complete removal of the *PGRP-LC* gene (Fig. 5c). Furthermore, overexpression of the regulatory isoform rPGRP-LC, but not of canonical PGRP-LC, blocked the effect of Rapa on age-related nuclear accumulation of Relish (Fig. 5e,f), suggesting rPGRP-LC as a driver of inflammaging in the fly fat body. This finding was surprising, as rPGRP-LC has been shown to have an anti-inflammatory function upon acute infection in young flies^61^. Consistent with this hypothesis, overexpression of rPGRP-LC did not affect nuclear localization of Relish in young flies (Fig. 5g). However, prolonged overexpression of rPGRP-LC induced accumulation of Relish in middle-aged flies, indicating that it can induce inflammation in an age-dependent manner. There was no additional effect of rPGRP-LC expression observed in old flies, probably due to the already very high level of Relish nuclear accumulation. Having identified rPGRP-LC as a driver of inflammaging, we next used a GFP-tagged rPGRP-LC protein to address its subcellular localization in fat body cells. Interestingly, GFP–rPGRP-LC protein accumulated in enlarged lysosomes induced by activation of S6K and Rapa treatment (Fig. 5h), suggesting reduced degradation by the endolysosome. These results suggest that inflammaging in flies is induced by rPGRP-LC and controlled by its endolysosome-dependent degradation.

### TORC1–S6K–Syx13 mediates inflammaging and immunosenescence

**Fig. 6:**
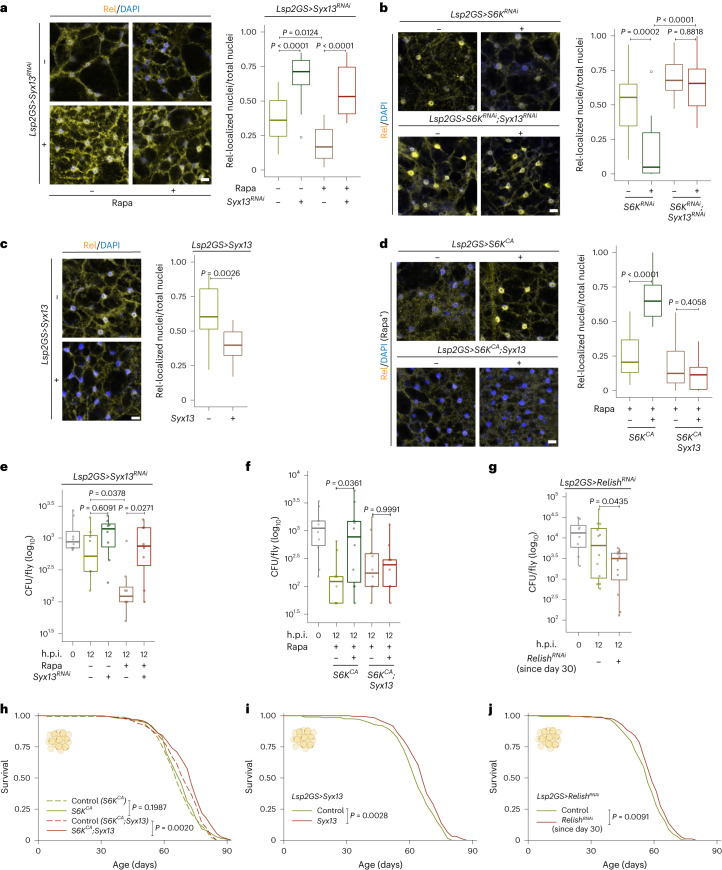
The TORC1–S6K–Syx13 axis regulates inflammaging, immunosenescence and lifespan. **a**,**b**, Knockdown of Syx13 blocked the effect of Rapa (**a**, Rapa: *P* = 0.0012, *Lsp2GS>Syx13*^*RNAi*^ induction: *P* = 1.026 × 10^−10^, interaction *P* = 0.2930; *n* = 14) and S6K knockdown (**b**, *Lsp2GS*>*S6K*^*RNAi*^ induction: *P* = 0.0004, *Lsp2GS>Syx13*^*RNAi*^ induction: *P* = 5.699 × 10^−7^, interaction *P* = 0.0088; *n* = 14) on Relish (Rel) nuclear localization in old fat body cells. **c**, Overexpression of Syx13 (*Lsp2GS>Syx13*; *n* = 14) repressed the age-related nuclear localization of Relish in old fat body cells. **d**, Overexpression of Syx13 (*Lsp2GS*>*S6K*^*CA*^*;Syx13*) rescued the effect of S6K activation on Relish localization (*Lsp2GS*>*S6K*^*CA*^ induction: *P* = 2.796 × 10^−5^, *Lsp2GS>Syx13* induction: *P* = 1.139 × 10^−10^, interaction *P* = 8.787 × 10^−9^; *n* = 14) in old flies treated with Rapa. **e**, Knockdown of Syx13 (*Lsp2GS>Syx13*^*RNAi*^) blocked the effect of Rapa on bacterial clearance, indicated by colony forming unit (CFU) assay (Rapa: *P* = 0.0093, *Lsp2GS>Syx13*^*RNAi*^ induction: *P* = 0.0057, interaction *P* = 0.2226; *n* = 8). **f**, Overexpression of Syx13 (*Lsp2GS*>*S6K*^*CA*^*;Syx13*) rescued the effect of S6K activation on bacterial clearance (*Lsp2GS*>*S6K*^*CA*^ induction: *P* = 0.0644, *Lsp2GS>Syx13* induction: *P* = 0.7042, interaction *P* = 0.0429; *n* = 10) in old flies treated with Rapa. **g**, Middle-age-onset repression of Relish using *Lsp2GS>Relish*^*RNAi*^ improved bacterial clearance in old flies (*n* = 8 in 0 h.p.i. group and *n* = 12 in 12 h.p.i. groups). **h**, Overexpression of Syx13 (*Lsp2GS*>*S6K*^*CA*^*;Syx13*) increased the lifespan of flies with S6K activation (*Lsp2GS*>*S6K*^*CA*^ induction: *P* = 0.1504, *Lsp2GS>Syx13* induction: *P* = 0.0148, interaction *P* = 0.2410; *n* = 200) **i**,**j**, Overexpression of Syx13 (**i**, *Lsp2GS>Syx13*; *n* = 200) and middle-age-onset repression of Relish (**j**, *Lsp2GS>Relish*^*RNAi*^; *n* = 200) extended lifespan. For box plot, the center is the median, the lower and upper bounds correspond to the first and third quartiles, the whiskers extend up to 1.5 times the interquartile range, and the minima and maxima are the observed minima and maxima. Each data point represents an average value per fat body (**a**–**d**) or per three whole flies (**e**–**g**). Two-sided linear mixed model (**a**–**d**) or two-sided two-way analysis of variance (ANOVA) with log transformation (**e** and **f**) followed by Tukey’s multiple comparison test. Two-sided Student’s *t*-test (**g**). CPH test and log-rank test (**h**–**j**). Representative survival curve of one (**h**) or two independent assay(s) (**i** and **j**). Scale bar, 10 μm.

Given the vital role of Syx13 in the endolysosomal system, we next investigated if Syx13 acts downstream of TORC1–S6K to regulate immune aging. Knockdown of Syx13 caused a robust induction in the number of Relish-positive nuclei in old fat bodies and blocked the effects of Rapa on Relish-positive nuclei and bacterial clearance (Fig. 6a,e). Relish repression upon S6K knockdown was also rescued by reducing Syx13 expression (Fig. 6b). Overexpression of Syx13 limited age-associated Relish activation and extended lifespan (Fig. 6c,i). Furthermore, while S6K activation blocked the effects of Rapa on Relish-positive nuclei, bacterial clearance and lifespan, co-overexpression of Syx13 restored the effects of Rapa (Fig. 6d,f,h and Extended Data Fig. 7c). Thus, Syx13 acts downstream of TORC1 and S6K to regulate inflammaging and immunosenescence. Despite the finding that autophagy can control inflammation and longevity^63^, blocking autophagy by Atg5-RNAi in the fat body did not block Rapa-dependent repression of Relish-positive nuclei and longevity (Extended Data Fig. 6d,e). Taken together, these findings show that TORC1–S6K–Syx13 signaling regulates immune aging via the endolysosomal system.

### Inflammaging in fly fat body is sex dependent

**Extended Data Fig. 9:**
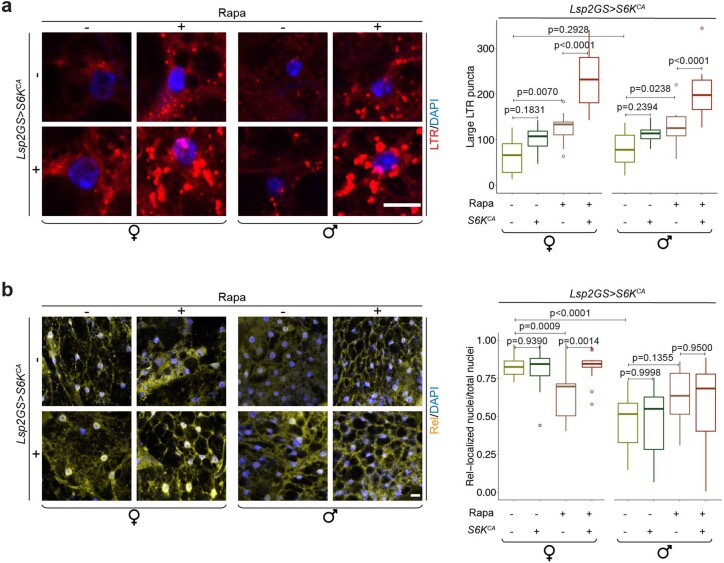
Reduced TORC1-S6K signalling lowers inflammageing and increases lifespan in female flies, but not in males. **a**, Fat body-specific adult-onset over-expression of constitutively active S6K induced enlarged lysosomes in response to rapamycin treatment in both females (rapamycin: p = 7.968e-10, *Lsp2GS* > *S6K*^*CA*^ induction: p = 5.785e-07, interaction p = 0.0053; n = 12) and males (rapamycin: p = 3.334e-07, *Lsp2GS* > *S6K*^*CA*^ induction: p = 4.931e-05, interaction p = 0.0786; n = 12). **b**, The aged fat body cells of male flies had lower age-related Relish localisation in comparison to female flies (n = 14). Rapamycin treatment suppressed the age- related nuclear localisation of Relish in old fat body cells of female flies. Overexpression of constitutive active S6K specifically in the fat body (*Lsp2GS* > *S6K*^*CA*^) blocked the effect of rapamycin on the age-related nuclear localisation of Relish in female flies (rapamycin: p = 0.0124, *Lsp2GS* > *S6K*^*CA*^ induction: p = 0.0213, interaction p = 0.0024; n = 14), but not in males (rapamycin: p = 0.0070, *Lsp2GS* > *S6K*^*CA*^ induction: p = 0.6618, interaction p = 0.7530; n = 14). For box plot, the centre is the median, the lower and upper bounds correspond to the first and third quartiles, the whiskers extend up to 1.5 times the interquartile range, and the minima and maxima are the observed minima and maxima. Each data point represents an average value per fat body. Two-sided linear mixed model for comparison between male and female control groups. Two-sided linear mixed model followed by Tukey’s multiple comparison test for comparison within each gender separately. Scale bar, 10 μm.

Knockout of S6K1 increased lifespan specifically in female but not in male mice^16^, suggesting a sex-specific role of S6K in the regulation of lifespan. Consistent with the mammalian finding, downregulation of S6K did also not increase lifespan in male flies (Extended Data Fig. 1b). To dissect the molecular mechanisms associated with the sex-specific effect of TORC1–S6K signaling, we first performed LysoTracker staining (Extended Data Fig. 9a). As in females, Rapa treatment also increased the number of LTR-positive punctae in fat bodies of males (Extended Data Fig. 9a). Furthermore, S6K activation in combination with Rapa treatment resulted in the accumulation of enlarged lysosomes in the fat body of both sexes (Extended Data Fig. 9a). Thus, changes in lysosomal morphology cannot explain the sex-specific lifespan difference. We next measured inflammation in old fat bodies of males and females (Extended Data Fig. 9b). Notably, old males had significantly less nuclear Relish than females, suggesting lower levels of inflammaging in the old male fat body, and neither Rapa treatment nor S6K activation affected nuclear localization of Relish, (Extended Data Fig. 9b). Thus, sex-specific differences in fat body inflammaging may contribute to the observed lifespan effects of S6K in males and females.

### Relish in fat bodies controls immunosenescence and longevity

Given that TORC1–S6K signaling regulated age-associated immune dysfunctions, we next addressed whether inflammaging in fat bodies affects pathogen clearance and longevity. Downregulating Relish level in fat bodies from middle-age on significantly improved bacterial clearance and extended female lifespan (Fig. 6g,j), demonstrating that ameliorating inflammaging may contribute to efficient clearance of bacteria at old age and lifespan extension.

### TORC1–S6K regulates Stx12 and immunoaging in mice

**Fig. 7:**
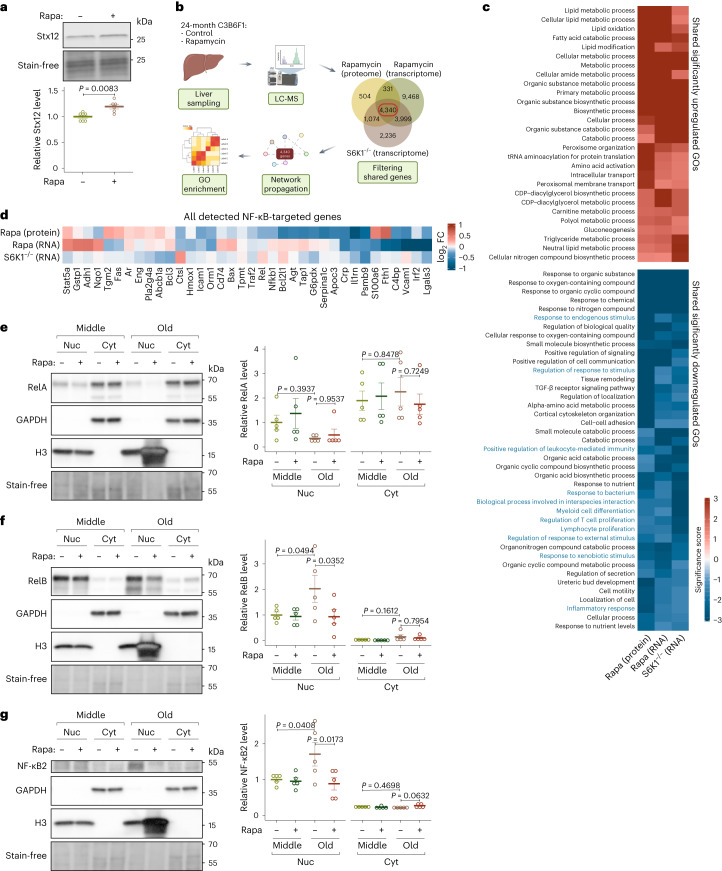
TORC1–S6K signaling regulates Stx12 expression and age-associated activation of the noncanonical NF-κB pathway in mouse liver. **a**, Rapa treatment increased Stx12 level in the liver of 12-month-old mice (*n* = 7). **b**, Schematic for the analysis workflow. Common genes present in all three datasets were used for network propagation and GO analysis. **c**, Only GO terms significantly regulated (*P* < 0.05, Fisher tests) in the same direction in all three datasets are shown. Immune-related annotations are shown in blue. Cells are colored by their log_10_ significance score. **d**, All NF-κB-targeted genes detected in all three datasets are shown. Cells are colored by their log_2_ fold change (FC). **e**, The effect of age and Rapa on the activity of the canonical NF-κB pathway as indicated by RelA/p65 level in liver subcellular fractions from 12-month-old (‘Middle’) and 24-month-old (‘Old’) mice (for nuclear fraction (‘Nuc’): age effect *P* = 0.0504, Rapa effect *P* = 0.4886, interaction *P* = 0.7559; for cytosolic fraction (‘Cyt’): age effect *P* = 0.9721, Rapa effect *P* = 0.7437, interaction *P* = 0.4936; *n* = 4). **f**,**g**, The effect of age and Rapa on the activity of the noncanonical NF-κB pathway as indicated by RelB level (**f**, for nuclear fraction (‘Nuc’): age effect *P* = 0.1050, Rapa effect *P* = 0.0680, interaction *P* = 0.0944; for cytosolic fraction (‘Cyt’): age effect *P* = 0.0310, Rapa effect *P* = 0.5382, interaction *P* = 0.8136; *n* = 4) and by NF-κB2/p52 level (**g**, for nuclear fraction (‘Nuc’): age effect *P* = 0.1218, Rapa effect *P* = 0.0405, interaction *P* = 0.0630; for cytosolic fraction (‘Cyt’): age effect *P* = 0.7063, Rapa effect *P* = 0.2032, interaction *P* = 0.0636; *n* = 4) in the liver subcellular fractions from 12-month-old (‘Middle’) and 24-month-old (‘Old’) mice. Data are displayed as mean ± s.e.m. Two-sided one-way analysis of variance (ANOVA) (**a**). Two-sided two-way ANOVA followed by Šídák’s multiple comparison test for comparison within nuclear fraction and cytosolic fraction separately (**e**–**g**).

Syx13 levels were upregulated in fly fat bodies upon downregulation of TORC1–S6K signaling (Fig. 3a,b). Interestingly, Syx13 levels were also upregulated in long-lived S6K mutant worms^28^ and in Rapa-treated yeast^64^, suggesting that this regulation is evolutionarily conserved, at least among yeast, worms and flies. In line with the findings in invertebrates, chronic Rapa treatment significantly increased Stx12 protein levels in the liver of female mice (Fig. 7a), suggesting that the TORC1-associated regulation of Stx12 is conserved from yeast to mammals.

To address whether reduced TORC1–S6K signaling also affected immunoaging in mice, we performed proteomics profiling of old liver samples from long-lived mice treated with Rapa (Supplementary Table 4) and integrated these data with previously published transcriptome analyses of livers of aged mice with Rapa treatment or S6K1 deficiency^16,65^ (Fig. 7b). Network propagation analysis^38^ of the 4,340 proteins shared between the three datasets identified immune-related processes, including inflammation and leukocyte proliferation, as commonly downregulated by S6K deficiency or Rapa treatment, while lipid- and translation-related processes were enhanced (Fig. 7c and Supplementary Table 5). In flies, the NF-κB-like transcription factor Relish was downregulated upon TORC1 repression in a S6K-dependent manner. Thus, we next explored the changes of NF-κB signaling in old liver. Consistent with the findings in *Drosophila*, NF-κB-target genes were generally repressed in old liver upon TORC1–S6K inhibition, including genes crucial for inflammatory responses, such as galectin-3 (*Lgals3*)^66^, interferon regulatory factor 2 (*Irf2*)^67^, proteasome subunit beta 9 (*Psmb9*)^68^, interleukin 1 receptor antagonist (*Il1rn*)^69^, C-reactive protein (*Crp*)^70^ and TNF receptor-associated factor 2 (*Traf2*)^71^ (Fig. 7d).

In mammals, activation of NF-κB signaling involves a canonical and noncanonical pathway branch that lead to the nuclear translocalization of the RelA/NF-κB1 dimer or the RelB/NF-κB2 dimer^72^, respectively. Nuclear/cytosol fractionation coupled with western blot showed that RelA, the canonical NF-κB transcription factor, was primarily localized in the cytosol and was unaffected by aging or Rapa (Fig. 7e). In contrast, the noncanonical NF-κB transcription factors, RelB and NF-κB2, were mainly in the nuclei of old liver and reduced by Rapa treatment (Fig. 7f,g), suggesting age-related activation of noncanonical NF-κB signaling in the mouse liver that can be counteracted by Rapa treatment.

## Discussion

**Extended Data Fig. 10:**
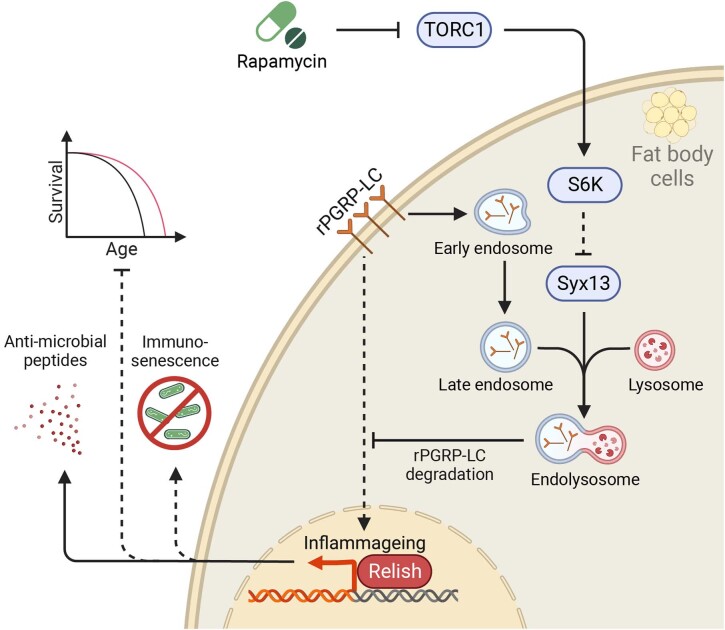
Schematic of the findings. S6K in the fly fat body plays a crucial role in rapamycin-related longevity. Mechanistically, S6K modulates the endolysosomal system by regulating the expression of the SNARE family protein Syx13. This regulation is vital for mitigating rPGRP-LC-induced inflammageing. The positive impact on inflammageing in the fly fat body leads to enhanced immune function and prolonged lifespan in aged flies.

Although S6K is a key downstream effector of mTOR signaling and has been implicated in determination of lifespan in invertebrates and mammals, the molecular and cellular mechanisms are still elusive. Here we show that, in *Drosophila*, lowered activity of S6K in the fat body is essential for mTOR-dependent longevity, and that it regulates endolysosomal morphology, inflammaging and immunosenescence in the aging fat body. Modifying endosome formation, but not autophagy, affected inflammaging by degrading rPGRP-LC, suggesting a causal link between endolysosome and inflammaging. We identified Syx13 as a molecular link that regulates endosome formation, inflammaging and lifespan downstream of TORC1–S6K signaling. We uncovered a considerable sexual dimorphism in fat body inflammaging, potentially explaining the different lifespan impacts of S6K observed in males and females. Furthermore, repression of the NF-κB-like IMD pathway in the fly fat body enhanced clearance of bacteria and extended lifespan. Importantly, long-term treatment with Rapa increased Stx12 levels in mouse liver, and alleviation of immune processes was a common denominator of TORC1–S6K inhibition in RNA and proteomics profiles from the liver of old Rapa-treated and S6K1 null mice. Furthermore, Rapa lowered age-associated activation of noncanonical NF-κB pathway in mouse liver, indicating that the effects of TORC1–S6K–Stx12 on immunoaging may be evolutionarily conserved from flies to mice. In summary, our findings highlight an important role for the TORC1–S6K–Syx13 signaling axis in inflammaging, immunosenescence and longevity (Extended Data Fig. 10).

Deletion of S6K increases lifespan in worms and of S6K1 does so in female mice^16,28^. In *Drosophila*, deletion of S6K is developmental lethal, but overexpression of a kinase-dead S6K has also been shown to extend adult lifespan^73^. However, whether this lifespan extension was caused by a loss in S6K activity or by titration of the Raptor protein, a key component of the TORC1 complex that directly interacts with S6K via the Raptor-binding TOS motif, was not clear^74^. We found that RNAi-mediated downregulation of S6K ubiquitously and in the fat body, but not in the intestine, neurons, muscle or heart extended lifespan to a similar extent, establishing the fat body as the key tissue for S6K-mediated longevity. Consistently, fat-body-specific overexpression of a constitutively active S6K protein blocked the effect of Rapa on longevity, demonstrating that lowered S6K activity in the fat body is essential for TORC1-mediated lifespan extension. In mice, phosphorylation of glutamyl-prolyl-tRNA synthetase (EPRS) has been linked to the effects of S6K1 deletion on adiposity and lifespan^8^. However, the *Drosophila* EPRS sequence lacks the mammalian EPRS phosphorylation site for S6K1. Furthermore, we did not find a role for S6K in Rapa-mediated lipid storage. S6K and autophagy are both essential for lifespan extension upon TORC1 inhibition^4^. However, while S6K function is required in the fat body but not in the gut, autophagy is essential in the gut and not the fat tissue^8^. Although the gut has recently been identified as a key organ in determining health and longevity^11,12,75^, our results suggest that the S6K-dependent phenotypes that we observed are independent of gut health.

Rapa treatment or the removal of S6K prolongs the lifespan of female animals more than males^4,16,32^, suggesting an evolutionarily conserved, sexually dimorphic aspect of longevity mechanisms. In line with the observation that genes related to the noncanonical NF-κB pathway are more abundant in the aged livers of female mice compared to male mice^76^, we have noted relatively higher levels of inflammaging in fat bodies of female flies compared to males, which can be further mitigated by inhibiting TORC1–S6K signaling. Given that suppressing inflammaging extended lifespan in female flies, the sex-specific age-prolonging effects of S6K may stem from its positive impact on inflammaging in female flies. Importantly, we found that S6K modulated lysosomal morphology, a key factor in both inflammaging and lifespan, in both sexes. This finding suggests that, while endolysosome-dependent regulation of inflammaging is crucial in female flies, male flies may possess distinct unknown mechanisms for controlling age-related inflammation.

Lysosomes are key to cellular proteostasis, and dysfunction of lysosomes contributes to age-associated pathologies^9^. Using LysoTracker staining and electron microscopy we observed a strong increase in lysosomal size associated with multilamellar structures in fat bodies expressing activated S6K and treated with Rapa. These changes are reminiscent of lysosomal storage diseases^47^, and may indicate impaired function caused by defects in lysosome or membrane fusion. These changes in the lysosome were rescued by overexpression of Syx13, a SNARE family protein that regulates autophagosome maturation and vesicle fusion^77,78^. Knockdown of Syx13 in human cells also induces multilamellar lysosome-like organelles^25^, suggesting evolutionary conservation of this function between flies and humans. Furthermore, Syx13 levels were upregulated in long-lived S6K mutant worms^28^ and also in Rapa-treated yeast^64^, flies and mice, suggesting that the regulation of Syx13 by TORC1 via S6K is evolutionarily conserved among these species. Protein expression of Syx13 has been shown to be positively regulated by short-term TORC1 inhibition upon neuronal injury or in mammalian cell culture^42,79,80^, possibly indicating a difference between acute stress and chronic inhibition of the mTOR pathway. Noteworthy, in contrast to Rapa feeding, Syx13 levels were not decreased upon activation of S6K under untreated control conditions and were even slightly upregulated, which might reflect a compensatory response upon S6K activation to counteract the detrimental effects of S6K on lysosome function.

Maintaining lysosomal function with age improves healthspan and lifespan in diverse animals^10,13,81–83^, effects so far mainly attributed to increased autophagy^9,84^. In contrast, we found that TORC1–S6K signaling regulates age-associated activity of the NF-κB-like transcription factor Relish and AMP expression via the endolysosomal system in the fat body, the main immune-responsive tissue of *Drosophila*. Age-associated inflammation could be caused by external factors like pathogen stimulation due to increased gut leakiness with age and/or intrinsic deterioration of fat body function^59,75^. The finding that Rapa repressed the number of Relish-positive nuclei and AMP expression even when bacterial growth was prevented by antibiotic treatment suggests that TORC1–S6K signaling regulates inflammaging via suppression of pathogen-independent sterile inflammation, which is in line with the findings in mammals^24,85^.

The endolysosomal system may directly affect the intracellular transport and degradation of immune regulators and receptors^61,62^. Here we discovered the importance of rPGRP-LC, a regulatory PGRP-LC isoform that helps resolve infection-induced IMD pathway activation via endosomes in young flies^62^, in age-associated inflammation. In middle-aged flies, rPGRP-LC might induce inflammation by an age-related decrease in internalization^86–88^. As a result, the rPGRP-LC–ligand complex, accumulated in the endolysosome, may not fully degrade and continues to activate the IMD pathway. Besides, similar to the endosomal sensing mechanisms of NOD2 (ref. ^89^), incomplete degradation of the rPGRP-LC–ligand complex may release its ligand, which is recognized by PGRP-LE, the intracellular receptor of the IMD pathway^53^. As Syx13 mediates endosome–lysosome fusion, overexpressing Syx13 could accelerate the internalization and degradation of the ligand and thereby suppress inflammaging. However, the source of PGRP-LC and rPGRP-LC ligands remains uncertain, especially when considering the role of PGRP-LC and S6K in inflammaging in the absence of bacterial infection. Thus, an alternative hypothesis suggests that rPGRP-LC accumulation in the cytosol may trigger other cytosolic stress mechanisms that could initiate inflammatory response, such as endoplasmic reticulum (ER) stress^90^. Given that Rapa suppresses ER stress and its associated apoptosis in vitro^91^, the involvement of rPGRP-LC and TORC1–S6K signaling in ER stress-associated inflammaging needs further investigation.

Age-associated increases of RelB and NF-κB2 have been reported in human lymphocytes^92^. Our data reveal that Rapa suppressed the age-associated activation of noncanonical NF-κB pathway in mouse liver. Given that the noncanonical NF-κB pathway is triggered by limited stimuli, it points to its specialized role in the immune system^72^. Understanding how the noncanonical NF-κB signaling is regulated in aging, especially considering the role of rPGRP-LC in the *Drosophila* NF-κB-like IMD pathway activation, could shed light on the root causes and the consequences of inflammaging in mammals.

Another common feature of the aged immune system is immunosenescence^56,57^. Manipulation of TORC1–S6K–Syx13 signaling in the fly fat body alleviated pathogen clearance, suggesting suppression of immunosenescence. Inhibition of TORC1 in older adults elevates type 1 interferon signaling, which mediates anti-virus immune response and improves immune function^93^. Interestingly, these interferon-induced genes include *IFITM3* (ref. ^94^) and *Mx1* (ref. ^95^), which are associated with endocytic trafficking. Thus, the endolysosomal system might also play a role in improved immune function in humans in response to TORC1 inhibition. Immunosenescence has long been considered to drive inflammaging^96^. However, ameliorating inflammaging by repressing the key inflammatory regulator Relish in the fly fat body was sufficient to ameliorate immunosenescence, suggesting that inflammaging in the fly fat body acts upstream of immunosenescence. Immunosenescence in flies is often caused by the decreased phagocytic activity of hemocytes at advanced age^59,97^. Since the immune response in the fat body controls hemocyte activation^98^, one potential hypothesis would be that TORC1–S6K–Syx13 signaling in the fat body regulates hemocyte aging by inter-tissue communication, thereby ameliorating immunosenescence. In addition to the hemocyte-related cellular immune response, the fly fat body secrets AMPs into the hemolymph to kill invading pathogens; however, the functional capacity of this humoral immune response declines with aging^99^. Regulation of TORC1–S6K–Syx13 signaling in the fat body hence may be important for mediating pathogen-induced AMP expression in old flies.

In summary, our results establish that TORC1–S6K–Syx13 signaling regulates inflammaging in hepatic tissues via the endolysosomal system, thereby alleviating immunosenescence and enhancing longevity.

## Methods

### Fly stock and husbandry

All transgenic fly lines were backcrossed for at least six generations into the outbred wild-type strain, white Dahomey (*w*^*Dah*^)^100^. For experiments, flies were maintained on 10% (w/v) brewer’s yeast, 5% (w/v) sucrose and 1.5% (w/v) agar food at 25 °C, 60% humidity, on a 12 h:12 h light:dark cycle. Rapa (LC Laboratories, #R-5000) was dissolved in ethanol and added to the food at a concentration of 200 µM. RU486 (Sigma, #M8046) was dissolved in ethanol and added to the food at a concentration of 100 µM to induce gene expression using the GeneSwitch system^101^. The corresponding control food contained only ethanol. Female flies were used in all experiments, with the exception of the experiments shown in Extended Data Fig. 1b and Extended Data Fig. 9. Fly stocks are listed in Supplementary Table 6. Fly genotypes are indicated in the figure legend or in the panel body.

### Mouse husbandry

The mouse Rapa study was performed in accordance with the recommendations and guidelines of the Federation of the European Laboratory Animal Science Association, with all protocols approved by the Landesamt für Natur, Umwelt und Verbraucherschutz, Nordrhein-Westfalen, Germany (reference no. 81-02.04.2019.A313). Female F_1_ hybrid mice (C3B6F1) were generated in-house by crossing C3H/HeOuJ females with C57BL/6NCrl males (strain codes 626 and 027, respectively, Charles River Laboratories). Animals were housed in groups of five females in individually ventilated cages under specific-pathogen-free conditions with constant temperature (21 °C), 50–60% humidity and a 12 h:12 h light:dark cycle. For environmental enrichment, mice had constant access to nesting material and chew sticks. Rapa treatment was initiated at 6 months of age and was administrated continuously. Encapsulated Rapa was obtained from Rapa Holdings and was added to the food (ssniff R/M-low phytoestrogen, ssniff Spezialdiäten) at a concentration of 42 mg kg^−1^. Control food contained corresponding amounts of the encapsulation material Eudragit S100. Both Rapa and control food contained 3.2 ml kg^−1^ PEG-400. For tissue collection, mice were killed by cervical dislocation, and tissues were rapidly collected and snap-frozen using liquid nitrogen.

### Generation of *an UAS-Syx13* transgenic fly line

To generate the transgenic *UAS-Syx13* fly line, full-length *Syx13* sequence was polymerase chain reaction (PCR) amplified with primers (forward: *TTTTTTCTCGAGCACCatgtccaaggccttgaaca* and reverse: *TTTTTTTCTAGAttaactgttcagtttggcaacga*) using complementary DNA clone LD27581 (Drosophila Genomics Resource Center) as template. The PCR product was digested with *XhoI* and *XbaI* (NEB) and cloned into the *pUAST-attB* vector^102^. To increase expression efficiency, *a CACC* Kozak sequence was inserted directly before the *ATG* start codon. The phiC31-mediated integrase system^102^ was used to generate transgenic flies, using the *attP40* insertion site^103^.

### Lifespan, fecundity and starvation assays

For lifespan assays, parental flies were allowed to lay eggs for 18 h, embryos were collected in phosphate-buffered saline (PBS) and dispensed into bottles. Flies that enclosed within a 24 h time window were then transferred to fresh bottles where they were allowed to mate for 48 h. Subsequently, flies were anesthetized with CO_2_ and 20 female flies were sorted to vials. Flies were transferred to fresh food vials every 2–3 days and scored for death. For fecundity assays, flies were treated as described above for the lifespan assay, but only five female flies were used per vials. Eggs laid within 20 h were collected and counted twice a week in the first four weeks. For starvation assays, flies were reared and maintained as for lifespan assay. Ten-day-old flies were transferred to 1% agar and scored for deaths at least twice per day.

### Peptide preparation for liquid chromatography–tandem mass spectrometry analysis

Dissected fat bodies from 10-day-old and 50-day-old female flies (five fat bodies were pooled as a replicate, four replicates per group) or 50 mg mouse livers were lysed in 6 M guanidinium chloride, 2.5 mM tris(2-carboxyethyl)phosphine, 10 mM chloroacetamide and 100 mM Tris–HCl for 10 min at 95 °C. Lysates were further disrupted by using a Bioruptor plus (Diagenode). Samples were then diluted with 20 mM Tris and trypsin (Trypsin Gold, Promega, #V5280, 1:200 w/w) and digested overnight at 37 °C. Digestion was stopped by adding formic acid (FA) to a final concentration of 1%. Peptide cleaning was performed by using in-house C18-SD (Empore) StageTips^104^. Therefore, StageTips were washed with methanol and 40% acetonitrile (ACN)/0.1% FA and finally equilibrated with 0.1% FA. Digested peptides were loaded on the equilibrated StageTips. StageTips were washed twice with 0.1% FA, and peptides were eluted with 40% ACN/0.1% FA and then dried in a Speed-Vac (Eppendorf) at 45 °C.

### TMT labeling and fractionation

Eluted peptides were reconstituted in 0.1 M triethylammonium bicarbonate. TMTpro 16plex (ThermoFisher, #A44522) labeling was carried out according to the manufacturer’s instruction with the following changes: 0.5 mg of TMT reagent was resuspended with 33 µl of anhydrous ACN. Seven microliters of TMT reagent in ACN was added to 9 µl of clean peptide in 0.1 M triethylammonium bicarbonate. The final ACN concentration was 43.75%, and the ratio of peptides to TMT reagent was 1:20. All 16 samples were labeled in one TMT batch. After 60 min of incubation the reaction was quenched with 2 µl of 5% hydroxylamine. Labeled peptides were pooled, dried, resuspended in 0.1% FA and desalted using StageTips. Samples were fractionated on a 1 mm × 150 mm, 130 Å, 1.7 µm ACQUITY UPLC Peptide CSH C18 Column (Waters, #186006935), using an Ultimate 3000 UHPLC (ThermoFisher). Peptides were separated at a flow of 30 µl min^−1^ with a 88 min segmented gradient from 1% to 50% buffer B for 85 min and from 50% to 95% buffer B for 3 min; buffer A was 5% ACN, 10 mM ammonium bicarbonate, buffer B was 80% ACN, 10 mM ammonium bicarbonate. Fractions were collected every 3 min, and fractions were pooled in two passes (1 + 17, 2 + 18 and so on) and dried in a Speed-Vac (Eppendorf).

### Liquid chromatography–tandem mass spectrometry analysis and protein identification

Dried fractions were resuspended in 0.1% FA and separated on a 500 mm, 0.075 mm Acclaim PepMap 100 C18 HPLC column (ThermoFisher, #164942) and analyzed on a Orbitrap Lumos Tribrid mass spectrometer (ThermoFisher) equipped with a FAIMS device (ThermoFisher) that was operated in two compensation voltages, −50 V and −70 V. Synchronous precursor selection based MS3 was used for TMT reporter ion signal measurements. Peptides were separated by EASY-nLC1200 using a 90 min linear gradient from 6% to 31% buffer; buffer A was 0.1% FA, buffer B was 0.1% FA, 80% ACN. The analytical column was operated at 50 °C. Raw files corresponding to the mouse livers were split on the basis of the FAIMS compensation voltage using FreeStyle (ThermoFisher). Fly fat body proteomics data were analyzed using Proteome Discoverer (version 2.4.1.15); mouse liver proteomics data were analyzed using MaxQuant (version 1.6.17.0). Isotope purity correction factors, provided by the manufacturer, were included in the analysis.

### Proteomics data analysis

Peptide intensity values were log_2_- and *z*-transformed. The results were rescaled by multiplying the *z*-intensity of each individual peptide with the global standard deviation of the log_2_-transformed data and adding back the global mean of the log_2_-transformed data. Only proteins that were detected in at least three out of the four replicates for each treatment were considered for downstream analyses. Missing values were imputed using the impute package (version 1.66.0) in R. Differential expression analysis was performed using the limma package (version 3.48.3) in R. For principal component analysis and plotting, batch effects from different dissection timepoints were removed from the normalized data using the limma package. The list of 5′TOP mRNA genes was obtained from Martin et al.^105^; the list of IMD-targeted AMPs was obtained from https://flybase.org/ using the Flybase ID: FBgg0001101; and the list of NF-κB-targeted genes was obtained from ref. ^106^.

### Network propagation

High-confidence interactions of the STRING protein–protein association network database for *Drosophila melanogaster* or *Mus musculus*^107^ were extracted as background for later propagation (combined score >899). Network propagation was performed using the BioNetSmooth package (version 1.0.0) in R. log_2_ fold changes of comparisons were imported into the network and propagated with *α* = 0.5 for 26 iterations (for flies) or 25 iterations (for mice). Top and bottom 5% proteins that contain more than four protein–protein interactions were used for further analysis.

### GO term enrichment

GO information was retrieved from org.Dm.eg.db package (version 3.13.0) or Uniprot-GOA database^108^ (version 2022-04-30) in R. The high-confidence interactions of STRING database (combined score >899) were set as background, and the results of network propagation were used for GO term enrichment analysis using Fisher tests with a minimal node size of five from ViSEAGO package (version 1.6.0) in R. Age-related GO terms were selected from the comparison of old versus young groups in the control treatment group of *Lsp2GS*>*S6K*^*CA*^ dataset after network propagation. For *Lsp2GS*>*S6K*^*CA*^ dataset, S6K^CA^-dependent Rapa-induced annotations were selected if the *P* value of Rapa versus control annotation is at least 100 times than the *P* value of *S6K*^*CA*^ versus *S6K*^*CA*^ + Rapa annotation (EtOHvsRapa.log10_pvalue − RUvsRURapa.log10_pvalue >2). S6K^CA^-independent Rapa-induced annotations were selected if the *P* value of Rapa versus control annotation is greater than 0.01 (EtOHvsRapa.log10_pvalue >2) and if the *P* value of Rapa versus control annotation is not 100 times than the *P* value of *S6K*^*CA*^ versus *S6K*^*CA*^ + Rapa annotation (EtOHvsRapa.log10_pvalue − RUvsRURapa.log10_pvalue ≤2). For mouse liver analysis, the biological processes significantly regulated (*P* < 0.05) as the same direction in all three datasets (hepatic proteome of 24m Rapa-treated mice, hepatic microarray of 25m Rapa-treated mice^65^, and hepatic microarray of 20m S6K1-deficient mice^16^) are plotted as shared up- or downregulated GO annotations after reducing redundancy via REVIGO (cutoff ‘0.7’, valueType ‘pvalue’, measure ‘SIMREL’). The significance score was calculated by the -log10_p-value with the sign of regulation direction (positive values for upregulated GOs and negative values for downregulated GOs).

### Electron microscopy

Fat bodies were fixed in 4% formaldehyde (Science Services) and 2.5% glutaraldehyde (Merck) in 0.1 M Cacodylate buffer (AppliChem) for 48 h at 4 °C. After washing in 0.1 M cacodylate buffer, tissues were treated with 2% osmiumtetroxid (Science Services) in 0.1 M Cacodylate buffer for 2 h. After dehydration of the sample with ascending ethanol concentrations followed by propylenoxid, samples were embedded in Epon (Sigma). Ultrathin sections (70 nm) were cut (EM-UC7, Leica Microsystems), collected onto mesh copper grids (Electron Microscopy Sciences) and contrasted with uranyl acetate (Plano GmbH) and lead citrate (Sigma). At least ten images per sample were acquired with a transmission electron microscope (JEM 2100 Plus, JEOL), a OneView 4K camera (Gatan) with DigitalMicrograph software at 80 kV at room temperature.

### LysoTracker staining

Tissues were dissected in PBS and stained with LysoTracker Red DND-99 (ThermoFisher, #L7528, 1:2,000) and Hoechst 33342 (Enzo, #ENZ-52401, 0.5 ng µl^−1^). Samples were rinsed with PBS and mounted with VECTASHIELD Antifade Mounting Medium (Vector Laboratories, #H-1000). Three images per sample were captured using a Leica TCS SP8 DLS confocal microscope with a 20× objective and 6× digital zoom-in. Images were processed by background subtraction, median filtering and spot quantification using Imaris 9 (Bitplane). Settings of the confocal microscope were kept consistent between images of an experiment. LysoTracker-positive puncta were defined as large, when the diameter was greater than 1.5 µm, which is the 75th percentile of the diameter of Rapa-induced LysoTracker-positive puncta.

### Immunofluorescence

Tissues were dissected in PBS and fixed 30 min with 4% formaldehyde, methanol-free (ThermoFisher, #28908). Samples were washed in 0.3% Triton-X/PBS (PBST), blocked in 5% bovine serum albumin (BSA)/PBST for 1 h at room temperature, incubated in primary antibody overnight at 4 °C, and in secondary antibody for 2 h at room temperature. The following primary antibodies were used: anti-Relish (Developmental Studies Hybridoma Bank, #21F3, 1:100), anti-Relish (RayBiotech, #RB-14-0004-200, 1:200) and anti-pH3 (Cell Signaling Technology, #9701, 1:200). The following secondary antibodies were used: Alexa Flour 488 goat anti-mouse IgG (ThermoFisher, #A11001, 1:1,000), Alexa Flour 633 goat anti-mice IgG (ThermoFisher, #A21050, 1:1,000), Alexa Flour 594 goat anti-rabbit IgG (ThermoFisher, #A11012, 1:1,000) and Alexa Flour 633 goat anti-rabbit IgG (ThermoFisher, #A21070, 1:1,000). Samples were mounted with VECTASHIELD Antifade Mounting Medium with DAPI (Vector Laboratories, #H-1200). Three images per sample were captured using a Leica TCS SP8 DLS confocal microscope with 20× objective and 6× digital zoom. For Relish protein localization, images were processed by background subtraction and median filtering using Imaris 9 (Bitplane). The number of nuclei with high Relish fluorescence intensity was normalized by the total number of nuclei. Confocal settings were kept consistent between images of the same experiment.

### Gut dysplasia assay

Guts were dissected in PBS and fixed 30 min with 4% formaldehyde, methanol-free (ThermoFisher, #28908). Samples were washed with PBS and mounted with VECTASHIELD Antifade Mounting Medium with DAPI (Vector Laboratories, #H-1200). The R2 region of the gut was captured using a Leica TCS SP8 DLS confocal microscope with a 40× objective. Images were analyzed using Fiji^109^ (US National Institutes of Health). The percentage of dysplasia was calculated as the ratio between the length of multilayer nuclei region and the total length of the R2 region of the gut.

### DQ–BSA/FITC–BSA pulse-chase assay

Fat bodies were dissected in Schneider’s *Drosophila* medium (Biowest, #L0207-500) and immediately pulsed for 10 min at room temperature with a mix of 100 µg ml^−1^ FITC–BSA (Sigma, #A9771) and 100 µg ml^−1^ DQ Red BSA (Life Technologies, #D12051) in Schneider’s *Drosophila* medium. Samples were rinsed with PBS and incubated for 2 h (chase) in Schneider’s *Drosophila* medium. Samples were rinsed with PBS and mounted with VECTASHIELD Antifade Mounting Medium (Vector Laboratories, #H-1000). Three images per sample were captured using a Leica TCS SP8 DLS confocal microscope with a 20× objective and 6× digital zoom-in. Settings of the confocal microscope were kept consistent between images of an experiment. FITC channel images were processed by background subtraction, median filtering and spot quantification using Imaris 9 (Bitplane). FITC-positive puncta were defined as lysosomes and the mean fluorescence intensity of DQ-Red within these lysosomes was quantified as a readout of lysosomal degradation capacity.

### Subcellular fractionation assay

Mouse livers were homogenized in ice-cold Nuclei EZ Prep lysis buffer (Sigma, #NUC-101) with 2 ml Dounce homogenizer, using both loose and tight pestles for 20 times. Extracts were incubated on ice for 30 min and centrifuged at 500*g* and 4 °C for 5 min. Nuclear and cytoplasmic fraction were collected separately and lysed and analyzed according to the immunoblotting assay. Histone H3 and GAPDH were used as nuclear and cytosolic marker proteins, respectively.

### Immunoblotting

Tissues were homogenized and lysed in ice-cold RIPA buffer supplemented with cOmplete, Mini, EDTA-free, Protease Inhibitor Cocktail (Roche, #11836170001) and PhosSTOP phosphatase inhibitor tablet (Roche, #049068370001) using a hand-held homogenizer. Extracts were centrifuged and protein concentrations were determined using Pierce BCA Protein Assay Kit (ThermoFisher, #23225). Extracts were mixed with 4× Laemmli loading buffer and boiled for 5 min at 95 °C. Ten micrograms (fly tissue and mouse tissue cell fractions) or 25 µg (mouse whole tissue lysis) of protein per lane on Any kD or 4–20% Criterion TGX stain-free precast gels (Bio-Rad) was transferred to Immobilon-FL polyvinylidene fluoride membrane (Millipore, #IPFL00010). Membranes were blocked by Intercept TBS Blocking Buffer (LI-COR, #927-60001) or 5% BSA for 1 h and probed with the following primary antibodies diluted in Intercept T20 TBS Antibody Diluent (LI-COR, #927-65001) or 5% BSA: anti-S6K^3^ (1:1,000), anti-pS6K T398 (Cell Signaling Technology, #9209, 1:1,000), anti-Relish (Developmental Studies Hybridoma Bank, #21F3, 1:100), anti-tubulin (Sigma, #T9026,1:5,000), anti-puromycin (Sigma, #MABE343, 1:1,000), anti-Stx12 (Synaptic Systems, #110 132, 1:1,000), anti-RelA (Cell Signaling Technology, #8242, 1:1,000), anti-RelB (Cell Signaling Technology, #4922, 1:1,000), anti-NF-κB2 (Cell Signaling Technology, #4882, 1:1,000), anti-GAPDH (Cell Signaling Technology, #2118, 1:5,000), anti-Histone H3 (abcam, #ab1791, 1:10,000). The following secondary antibodies were used: IRDye 800CW Goat anti-Mouse IgG (H + L) (LI-COR, #926-32210, 1:15,000), IRDye 680RD Goat anti-Rabbit IgG (H + L) (LI-COR, #926-68071, 1:15,000), HRP-conjugate Goat anti-Rabbit IgG Antibody (Sigma, #12-348; 1:10,000) and HRP-conjugate Goat anti-Mouse IgG Antibody (Sigma, #12-349; 1:10,000). Total protein on the membrane was visualized as stain-free signal using ChemiDoc MP Imagers (Bio-Rad) and was used for normalizing protein expression levels. Signal was developed using Amersham ECL Select Western Blotting Detection Reagent (GE Healthcare). Immunoblotting images were captured using Odyssey Infrared Imaging system with application software V3.0.30 (LI-COR) or using ChemiDoc MP Imagers with Image Lab V6.1.0.07 (Bio-Rad) and were analyzed using Fiji^109^ (US National Institutes of Health).

### RNA isolation and qRT–PCR

Total RNA was extracted from tissues using the RNeasy Mini Kit (Qiagen, #74106). RNA concentration was measured using the Qubit RNA Broad Range Assay Kit (ThermoFisher, #Q10211). cDNA synthesis was performed using SuperScript VILO Master Mix (ThermoFisher, #11755-250) with 600 ng total RNA as input. Quantitative reverse transcription (qRT)–PCR was performed using SYBR Green Master Mix (ThermoFisher, #4367659) on a QuantStudio6 Flex Real-Time PCR System (ThermoFisher). Relative expression was determined using the ∆∆CT method and *Rpl32* as normalization control. The following primers were used:

DptA-forward: CCACGAGATTGGACTGAATG

DptA-reverse: GGTGTAGGTGCTTCCCACTT

S6K-forward: ACTGGGCGCTCTCATGTTTG

S6K-reverse: TTGGCTTTCAGAATGGTCT

Rpl32-forward: ATATGCTAAGCTGTCGCACAAATGG

Rpl32-reverse: GATCCGTAACCGATGTTGGGCA

### Triglyceride assay

Five whole flies were homogenized by 0.05% Tween-20 and incubated at 70 °C for 5 min. Extracts were centrifuged, the supernatant was mixed with prewarmed Infinity Triglycerides reagent (ThermoFisher, #TR22421) and incubated at 37 °C for 5 min. Protein content was determined by Pierce BCA Protein Assay Kit (ThermoFisher, #23225). Triglyceride levels were determined by plate reader at the absorbance of 540 nm using a triglyceride standard (Sigma, #17811-1AMP). Protein levels were used for normalization.

### Puromycin incorporation assay

Fat bodies were dissected in PBS and immediately transferred to Schneider’s *Drosophila* medium with 5 µg ml^−1^ puromycin (Sigma, #P8833). For negative control, no puromycin was added in the Schneider’s *Drosophila* medium. Sample were incubated for 45 min with 400 rpm at 25 °C and then lysed according to the immunoblotting assay.

### Antibiotic treatment

For antibiotic treatment, tetracycline and ampicillin were dissolved in water and added to the food in a concentration of 100 mg l^−1^ and 50 mg l^−1^, respectively. To test whether the treatment was efficient at removing bacteria, flies were dipped in 70% ethanol for 3 min and rinsed with sterile PBS. Individual flies were mashed in sterile PBS. Samples were plated on lysogeny broth (LB) plates and cultured at room temperature, and the number of bacterial colonies was scored at day 7.

### Infection assays

For infection assays, flies were infected by pricking the thorax with a fine needle dipped in freshly cultured *Ecc15* (OD_600_200). At 0 or 12 h post-infection (h.p.i.), flies were surface sterilized with 70% ethanol, rinsed with sterile PBS, and then three flies were homogenized in sterile PBS. Tenfold-series dilutions of fly homogenates were plated on LB plates as spots, cultured at room temperature, and the number of bacterial colonies was scored the next day. ‘0 h.p.i.’ represented the initial infectious dose and was not included in the statistical analysis. For ex vivo infection assay, fat bodies were dissected in sterile PBS and immediately incubated with PBS containing freshly cultured *Ecc15* (final concentration OD_600_0.2) at room temperature for 1 h. For sham control, tissues were incubated with sterile PBS at room temperature for 1 h. Samples were then processed as described for immunofluorescence assay.

### Statistics and reproducibility

All statistical analyses were performed in GraphPad Prism 9 and R 4.1.0. Linear mixed model and negative binomial generalized linear model were generated and analyzed in R using lme4, lmertest and emmeans package. Proteomics data were analyzed in R using impute, limma, ViSEAGO package. To avoid batch effect of TMTpro 16plex labeling, we allocated distinct TMTpro 16plex channels to each biological replicate. Furthermore, batch effects arising from varying dissection timepoints were removed using removeBatchEffect function from limma package. Cox proportional hazards (CPH) test was performed in R using survival package. log-rank tests were performed in Microsoft Excel for Mac (version 16.62) and R using survival package. For small sample sizes (*n* ≤ 10), data are presented as individual points with mean ± standard error of the mean (s.e.m.). For large sample sizes (*n* > 10), box plots were used and the center is the median, the lower and upper bounds correspond to the first and third quartiles, the whiskers extend up to 1.5 times the interquartile range, and the minima and maxima are the observed minima and maxima. A small number of confocal microscopy images were excluded from analysis due to issues such as low signal-to-noise ratio, poor contrast, presence of artifacts, compromised specimen integrity, and saturation. These quality deficits hindered reliable data interpretation. No statistical methods were used to predetermine sample sizes, but our sample sizes are similar to those reported in previous publications^12,31,110^. Data distribution was assumed to be normal, but this was not formally tested. Sample sizes and statistical tests used are indicated in the figure legend. Flies of the same genotype were randomly assigned to vials containing different types of food for the subsequent experiments. All experiments were performed blind except for lifespan assays, proteomics data analysis, immunoblotting and qRT–PCR, which required documentation of fly genotype and crossing by the investigators.

### Reporting summary

Further information on research design is available in the Nature Portfolio Reporting Summary linked to this article.

### Supplementary information

Reporting Summary

Supplementary Table 1 List of all proteins identified by the proteomic analysis in young (day 10) and old (day 50) fat bodies of *Lsp2GS>S6K*^*CA*^ or *Lsp2GS>S6K*^*RNAi*^ flies.

Supplementary Table 2 List of differentially regulated proteins upon modified TORC1–S6K signaling. TORC1–S6K-dependent proteins were defined as proteins that were significantly changed (*P* < 0.05) by both S6K knockdown and Rapa treatment but that were not altered by Rapa treatment under S6K activation condition (*P* ≥ 0.05).

Supplementary Table 3 List of age-associated GO terms, Rapa-induced, S6K-dependent GO terms, and S6K inhibition-induced GO terms. Results contain three subontologies: biological process (BP), cellular component (CC) and molecular function (MF).

Supplementary Table 4 List of proteins identified by the proteomic analysis in the liver of 24-month-old Rapa-treated female mice.

Supplementary Table 5 List of GO terms aggregated across datasets (rapamycin-proteome, rapamycin-transcriptome and S6K1^−/−^-transcriptome). Common genes that have been detected in all three datasets were used for network propagation and GO analysis. Only functions significantly regulated (*P* < 0.05) in the same direction in all three datasets are shown.

Supplementary Table 6 List of fly strains used in this study.

### Source data

Statistical Source Data A single Excel file containing all statistical source data, with clearly named tabs for each Figure/Extended Data Figure item.

Uncropped Blots A single PDF file containing all supporting blots, with clearly labeled blots for each Figure/Extended Figure item.

## Data Availability

All data that support the findings of this study are available from the corresponding authors upon reasonable request. Source data are provided with this paper. The list of 5′TOP mRNA genes were obtained from Martin et al.^105^; the list of IMD-targeted AMPs was obtained from https://flybase.org/ using the Flybase ID FBgg0001101; and the list of NF-κB-targeted genes was obtained from ref. ^106^. The STRING V11 resource^107^ is available at https://string-db.org/. GO information was retrieved from org.Dm.eg.db package (version 3.13.0) or Uniprot-GOA database^108^ (version 2022-04-30) in R. The mass spectrometry proteomics data have been deposited to the ProteomeXchange Consortium via the PRIDE^111^ partner repository with the dataset identifier PXD035293.
