# Supplementary material for: Inhibition of S6K lowers age-related inflammation and increases lifespan through the endolysosomal system
Source: Nat Aging. 2024 Feb 27;4(4):491–509. doi: 10.1038/s43587-024-00578-3 (PMC11031405; doi:10.1038/s43587-024-00578-3)
Supplement: Supplementary file 1 — Reporting Summary [file 43587_2024_578_MOESM1_ESM.pdf]

nature portfolio

Corresponding author(s): Sebastian Grönke and Linda Partridge

Last updated by author(s): Jan 18, 2024

Reporting Summary

Nature Portfolio wishes to improve the reproducibility of the work that we publish. This form provides structure for consistency and transparency in reporting. For further information on Nature Portfolio policies, see our Editorial Policies and the Editorial Policy Checklist.

Statistics

For all statistical analyses, confirm that the following items are present in the figure legend, table legend, main text, or Methods section.

n/a	Confirmed	
☐	☒	The exact sample size (*n*) for each experimental group/condition, given as a discrete number and unit of measurement
☐	☒	A statement on whether measurements were taken from distinct samples or whether the same sample was measured repeatedly
☐	☒	The statistical test(s) used AND whether they are one- or two-sided<br>*Only common tests should be described solely by name; describe more complex techniques in the Methods section.*
☐	☒	A description of all covariates tested
☐	☒	A description of any assumptions or corrections, such as tests of normality and adjustment for multiple comparisons
☐	☒	A full description of the statistical parameters including central tendency (e.g. means) or other basic estimates (e.g. regression coefficient) AND variation (e.g. standard deviation) or associated estimates of uncertainty (e.g. confidence intervals)
☐	☒	For null hypothesis testing, the test statistic (e.g. *F*, *t*, *r*) with confidence intervals, effect sizes, degrees of freedom and *P* value noted<br>*Give P values as exact values whenever suitable.*
☒	☐	For Bayesian analysis, information on the choice of priors and Markov chain Monte Carlo settings
☐	☒	For hierarchical and complex designs, identification of the appropriate level for tests and full reporting of outcomes
☒	☐	Estimates of effect sizes (e.g. Cohen's *d*, Pearson's *r*), indicating how they were calculated

Our web collection on statistics for biologists contains articles on many of the points above.

Software and code

Policy information about availability of computer code

Data collection	qRT-PCR: QuantStudio Real-Time PCR Software v1.1<br>Immunoblotting: Image Lab version 6.1.0.07, Odyssey Infrared Imaging System, Application software version 3.0.30<br>Image acquisition: Leica Application Suite X version 3.x; DigitalMicrograph software version 3.x<br>Data collection: org.Dm.eg.db package (version 3.13.0); Uniprot-GOA database (http://www.ebi.ac.uk/GOA/, version 2022-04-30); the list of 5' TOP mRNA genes were obtained from Martin, et al. 2022; the list of IMD-targeted AMPs was obtained from https://flybase.org/ using the Flybase ID: FBgg0001101; and the list of NF-kB-targeted genes was obtained from https://bioinfo.lifl.fr/NF-KB/.
Data analysis	Microsoft Excel for Mac version 16.x was used for general data preparation<br>Fly fat body proteomics data were analysed using Proteome Discoverer (version 2.4.1.15); mouse liver proteomics data were analysed using MaxQuant (version 1.6.17.0).<br>Images were analysed using Fiji 2.3.0 and Imaris 9.<br>All statistical analyses were performed in GraphPad Prism 9 and R 4.1.0 (packages used: lme4, lmertest, emmeans; survival; limma; impute).<br>Network propagation and gene ontology analyses were performed using R 4.1.0 (packages: BioNetSmooth; ViSEAGO) .

For manuscripts utilizing custom algorithms or software that are central to the research but not yet described in published literature, software must be made available to editors and reviewers. We strongly encourage code deposition in a community repository (e.g. GitHub). See the Nature Portfolio guidelines for submitting code & software for further information.

Data

Policy information about availability of data

All manuscripts must include a data availability statement. This statement should provide the following information, where applicable:

- Accession codes, unique identifiers, or web links for publicly available datasets
- A description of any restrictions on data availability
- For clinical datasets or third party data, please ensure that the statement adheres to our policy

All data that support the findings of this study are available from the corresponding authors upon reasonable request. Source data are provided with this paper. The list of 5' TOP mRNA genes were obtained from Martin, et al. 106; the list of IMD-targeted AMPs was obtained from https://flybase.org/ using the Flybase ID: FBgg0001101; and the list of NF-kB-targeted genes was obtained from https://bioinfo.lifl.fr/NF-KB/. The STRING V11 resource107 is available at https://string-db.org/. Gene ontology information was retrieved from org.Dm.eg.db package (version 3.13.0) or Uniprot-GOA database (http://www.ebi.ac.uk/GOA/, version 2022-04-30) in R. The mass spectrometry proteomics data have been deposited to the ProteomeXchange Consortium via the PRIDE110 partner repository with the dataset identifier PXD035293.

Human research participants

Policy information about studies involving human research participants and Sex and Gender in Research.

Reporting on sex and gender	N/A
Population characteristics	N/A
Recruitment	N/A
Ethics oversight	N/A

Note that full information on the approval of the study protocol must also be provided in the manuscript.

Field-specific reporting

Please select the one below that is the best fit for your research. If you are not sure, read the appropriate sections before making your selection.

☒ Life sciences ☐ Behavioural & social sciences ☐ Ecological, evolutionary & environmental sciences

For a reference copy of the document with all sections, see nature.com/documents/nr-reporting-summary-flat.pdf

Life sciences study design

All studies must disclose on these points even when the disclosure is negative.

Sample size	No statistical methods were used to pre-determine sample sizes but our sample sizes are similar to those reported in previous publications (Filer et al., 2017, Nature; Lu et al., 2021, eLife; Lu et al., 2021, Nature Aging)
Data exclusions	A small number of confocal microscopy images were excluded from analysis due to issues such as low signal-to-noise ratio, poor contrast, presence of artifacts, compromised specimen integrity, and saturation. These quality deficits hindered reliable data interpretation.
Replication	All data presented were from independent biological replicates or independent experiments. All attempts at replication were successful. For lifespan assays, there were no attempts to replicate negative results, i.e., no lifespan extension upon S6K RNAi in the gut, muscle, heart tube and neuron. When lifespan extension was observed, this was confirmed in at least one independent experiment. For quantification experiments with confocal microscopy, at least 9 biological replicates were included. For localization experiments with confocal microscopy, at least 4 biological replicates were included. For quantification experiments with electron microscopy, at least 5 biological replicates were included. For other experiments, at least 3 biological replicates were included. Each replicate includes at least five fly tissues or was extract from independent mouse liver. The exact replicate number was indicated in the corresponding figure legends.
Randomization	Samples were allocated to groups/treatments randomly. Steps were taken to avoid batch effects. To avoid batch effect of TMTpro 16plex labelling, we allocated distinct TMTpro 16plex channels to each biological replicate. Furthermore, batch effects arising from varying dissection timepoints were removed using removeBatchEffect function from limma package.
Blinding	Blinding was performed wherever possible during sample preparation and analysis, including for the analysis of confocal and electron microscopy images. For other experiments, the investigators were not blinded to group allocations due to the fact that the genotypes of flies, as well as their crossing, needed to be carefully documented by the investigators.

Reporting for specific materials, systems and methods

We require information from authors about some types of materials, experimental systems and methods used in many studies. Here, indicate whether each material, system or method listed is relevant to your study. If you are not sure if a list item applies to your research, read the appropriate section before selecting a response.

Materials & experimental systems

n/a	Involved in the study
☐	☒ Antibodies
☒	☐ Eukaryotic cell lines
☒	☐ Palaeontology and archaeology
☐	☒ Animals and other organisms
☒	☐ Clinical data
☒	☐ Dual use research of concern

Methods

n/a	Involved in the study
☒	☐ ChIP-seq
☒	☐ Flow cytometry
☒	☐ MRI-based neuroimaging

Antibodies

Antibodies used

Primary antibodies: anti-S6K (home-made), anti-pS6K T398 (Cell Signaling Technology, #9209), anti-tubulin (Sigma, #T9026), anti-puromycin (Sigma #MABE343), anti-Stx12 (Synaptic Systems #110 132), anti-Relish (Developmental Studies Hybridoma Bank, #21F3), anti-Relish (RayBiotech, #RB-14-0004-200), anti-RelA (Cell Signaling Technology, #8242), anti-RelB (Cell Signaling Technology, #4922), anti-NF-kB2 (Cell Signaling Technology, #4882), anti-GAPDH (Cell Signaling Technology, #2118), anti-Histone H3 (abcam, #ab1791), anti-phospho-Histone H3 (Cell Signaling Technology, #9701)

Secondary antibodies: IRDye 800CW Goat anti-Mouse IgG (H + L) (LI-COR, #926-32210), IRDye 680RD Goat anti-Rabbit IgG (H + L) (LI-COR, #926-68071), HRP-conjugate Goat anti-Rabbit IgG Antibody (Sigma, #12-348), HRP-conjugate Goat anti-Mouse IgG Antibody (Sigma, #12-349), Alexa Flour 488 goat anti-mouse IgG (ThermoFisher, #A11001), Alexa Flour 633 goat anti-mice IgG (ThermoFisher, #A21050), Alexa Flour 594 goat anti-rabbit IgG (ThermoFisher, #A11012), Alexa Flour 633 goat anti-rabbit IgG (ThermoFisher, #A21070)

Validation

Anti S6K rabbit polyclonal, home-made. Validated by previous colleagues and published:
Slack C, Alic N, Foley A, Cabecinha M, Hoddinott MP, Partridge L. The Ras-Erk-ETS-Signaling Pathway Is a Drug Target for Longevity. Cell. 2015;162(1):72-83. doi:10.1016/j.cell.2015.06.023

Anti-pS6K T398 (Cell Signaling Technology, #9209) cited by 135 publications (https://www.cellsignal.com/products/primary-antibodies/phospho-drosophila-p70-s6-kinase-thr398-antibody/9209).

Anti-tubulin (Sigma, #T9026) cited by 4203 publications (https://www.citeab.com/antibodies/2304939-t9026-monoclonal-anti-alpha-tubulin-antibody-produce).

Anti-puromycin (Sigma #MABE343) cited by 513 publications (https://www.citeab.com/antibodies/1473291-mabe343-anti-puromycin-antibody-clone-12d10).

Anti-Stx12 (Synaptic Systems #110 132) has been verified by RNAi-based knockdown assay (https://doi.org/10.1242/jcs.196931). Cited by 20 publications (https://www.sysy.com/product/110132).

Anti-Relish (Developmental Studies Hybridoma Bank, #21F3) has been verified by null mutant and LPS stimulation assay (https://doi.org/10.1093/embo-reports/kvd072). Cited by 6 publications (https://www.citeab.com/antibodies/150578-anti-relish-c-21f3-relish-nf-kappab).

Anti-Relish (RayBiotech, #RB-14-0004-200) has validation via infection assay from the company's website (https://www.raybiotech.com/rabbit-anti-relish-en-2/). Cited by 2 publications (https://www.raybiotech.com/rabbit-anti-relish-en-2/).

anti-RelA (Cell Signaling Technology, #8242) cited by 4333 (https://www.cellsignal.com/products/primary-antibodies/nf-kb-p65-d14e12-xp-rabbit-mab/8242)

anti-RelB (Cell Signaling Technology, #4922) cited by 81 (https://www.cellsignal.com/products/primary-antibodies/relb-c1e4-rabbit-mab/4922)

anti-NF-kB2 (Cell Signaling Technology, #4882) cited by 281 (https://www.cellsignal.com/products/primary-antibodies/nf-kb2-p100-p52-antibody/4882)

anti-GAPDH (Cell Signaling Technology, #2118) cited by 7616 (https://www.cellsignal.com/products/primary-antibodies/gapdh-14c10-rabbit-mab/2118)

anti-Histone H3 (abcam, #ab1791) cited by 4330 (https://www.abcam.com/products/primary-antibodies/histone-h3-antibody-nuclear-marker-and-chip-grade-ab1791.html)

anti-phospho-Histone H3 (Cell Signaling Technology, #9701) cited by 1098 (https://www.cellsignal.com/products/primary-antibodies/

	phospho-histone-h3-ser10-antibody/9701)

Animals and other research organisms

Policy information about studies involving animals; ARRIVE guidelines recommended for reporting animal research, and Sex and Gender in Research

Laboratory animals	The 12-month-old and 24-month-old genetically heterogeneous C3B6F1 female mice (a cross between C3H/HeN female and C57Bl/6N male mice, obtained from Charles River Laboratories).<br>The 10-day-old, 30-day-old and 50-day-old Drosophila melanogaster were used. The fly strain, sex, and age information were described in detail in Methods and also in relevant figure legend or body. The following fly strains were used:<br>wDah<br>wDah;Lsp2GS<br>wDah;daGS<br>wDah;ElavGS<br>wDah;MHCGS<br>wDah;HandGS<br>wDah;TiGS<br>wDah;UAS-S6KCA<br>wDah;UAS-S6KRNAi<br>wDah;UAS-Atg5RNAi<br>wDah;UAS-Syx13<br>wDah;UAS-Syx13<br>wDah;UAS-Syx13RNAi<br>wDah;UAS-YFP-Rab5DN<br>wDah;UAS-YFP-Rab7DN<br>wDah;UAS-YFP-Rab5<br>wDah;UAS-YFP-Rab7<br>wDah;UAS-GFP-Lamp1<br>wDah;Lamp1-3xmCherry<br>wDah;UAS-RelRNAi<br>wDah;UAS-MitfRNAi<br>w; PGRP-LCΔE12<br>yw; UAS-PGRP-LCx (named as: UAS-LCx)<br>w; UAS-GFP-rPGRP-LCx (named as: UAS-GFP-rLCx)<br>w; GFP-PGRP-LC; PGRP-LCΔE12 (named as: resc(LCwt))<br>w; GFP-PGRP-LCΔex5; PGRP-LCΔE12 (named as: resc(LCΔex5))
Wild animals	Our study did not involve any wild animals.
Reporting on sex	Sex was reported in Methods and also in relevant figure legend or body.
Field-collected samples	Our study did not involve any field-collected samples.
Ethics oversight	The mouse rapamycin study was performed in accordance with the recommendations and guidelines of the Federation of the European Laboratory Animal Science Association (FELASA), with all protocols approved by the Landesamt für Natur, Umwelt und Verbraucherschutz, Nordrhein-Westfalen, Germany (reference no. 81-02.04.2019.A313).

Note that full information on the approval of the study protocol must also be provided in the manuscript.
