# Supplementary figures and images for: Inhibition of S6K lowers age-related inflammation and increases lifespan through the endolysosomal system
Source: Nat Aging. 2024 Feb 27;4(4):491–509. doi: 10.1038/s43587-024-00578-3 (PMC11031405; doi:10.1038/s43587-024-00578-3)

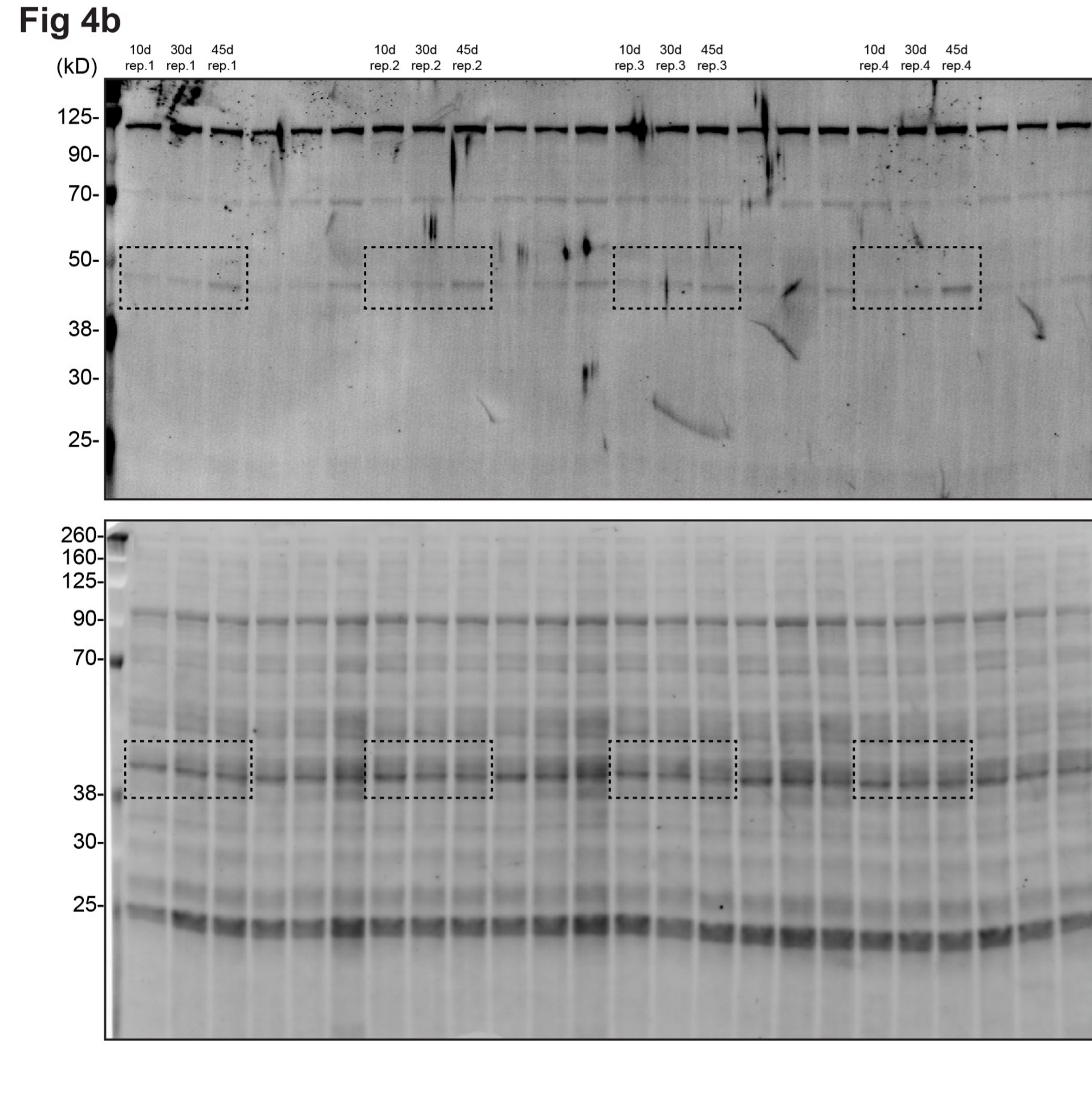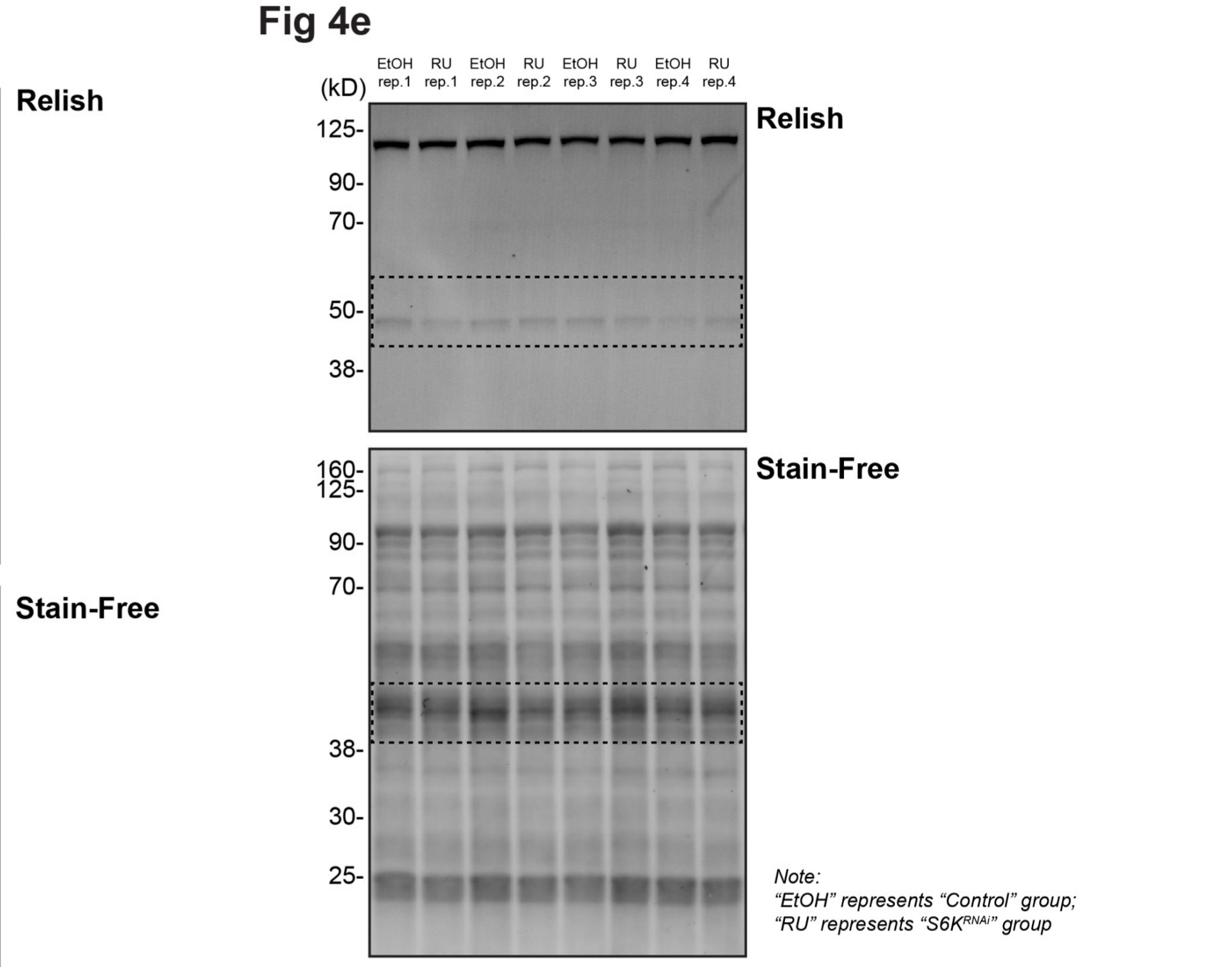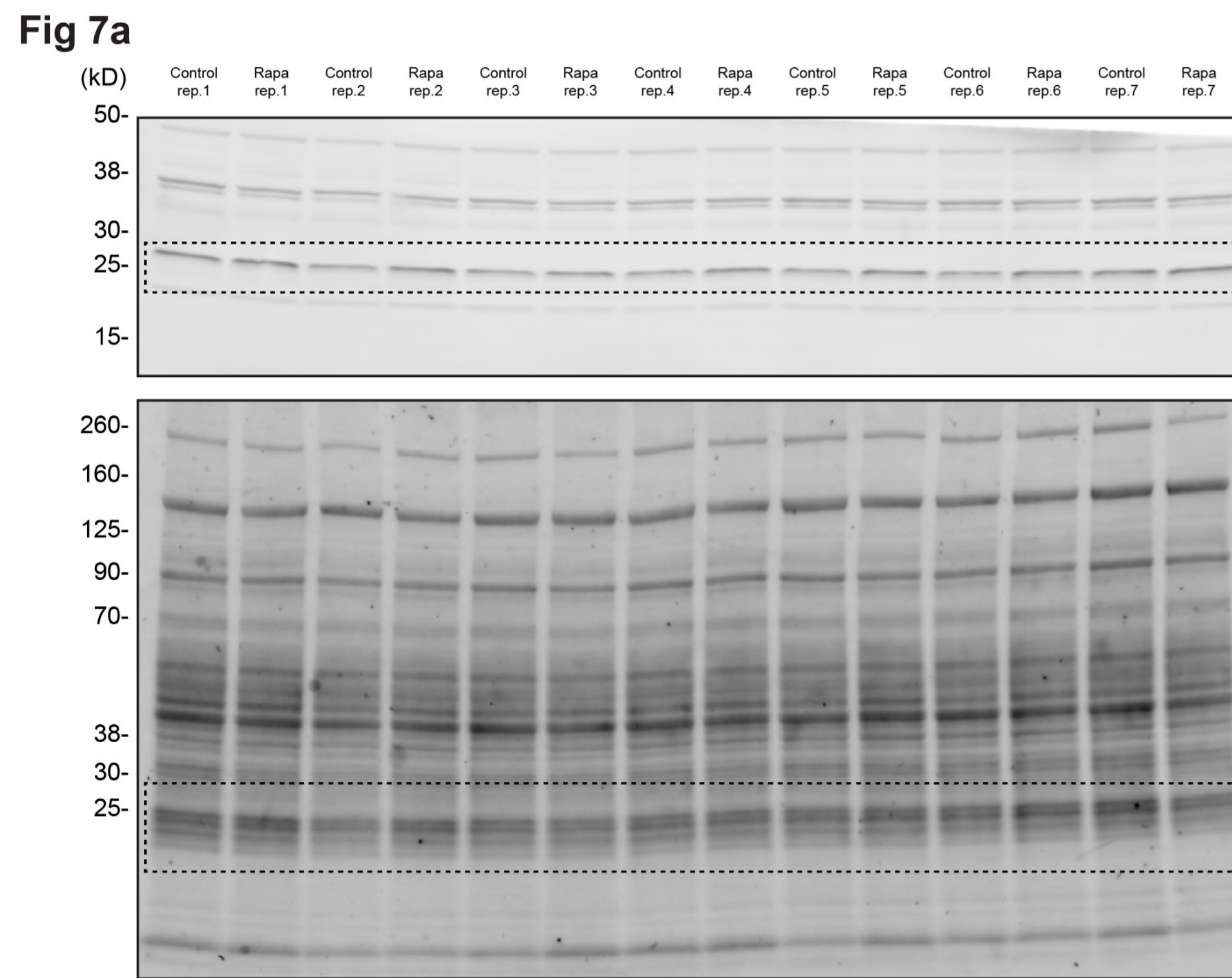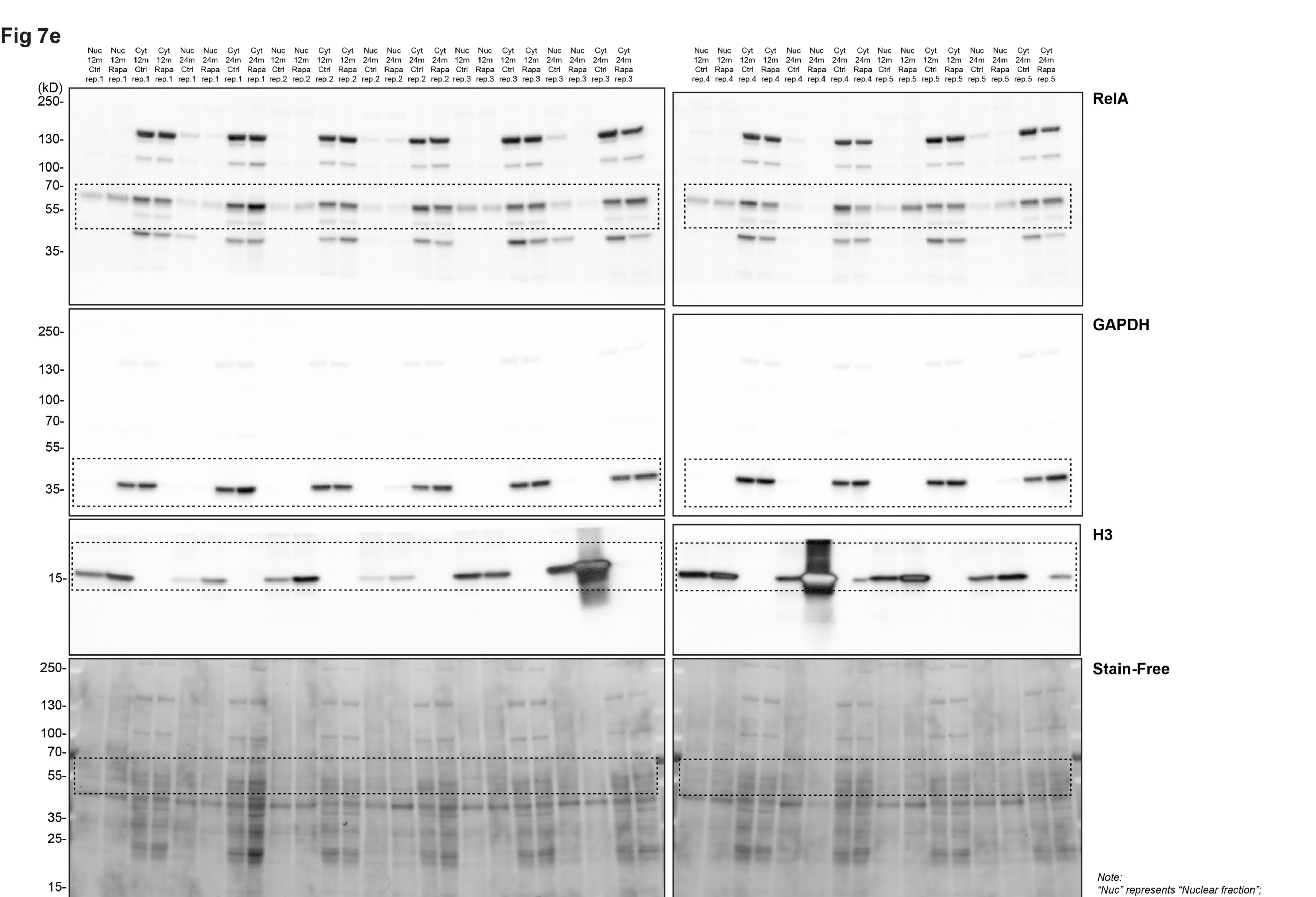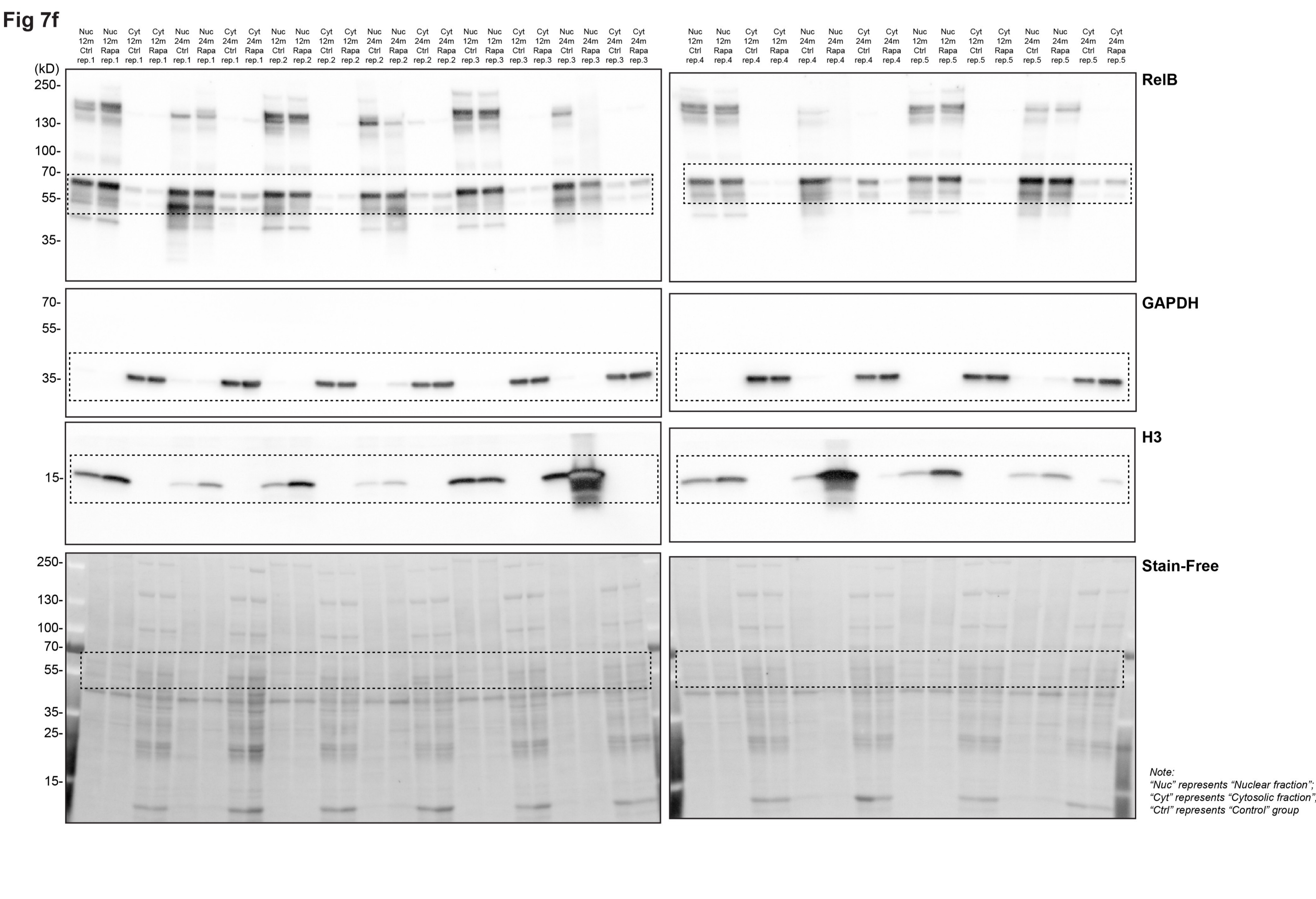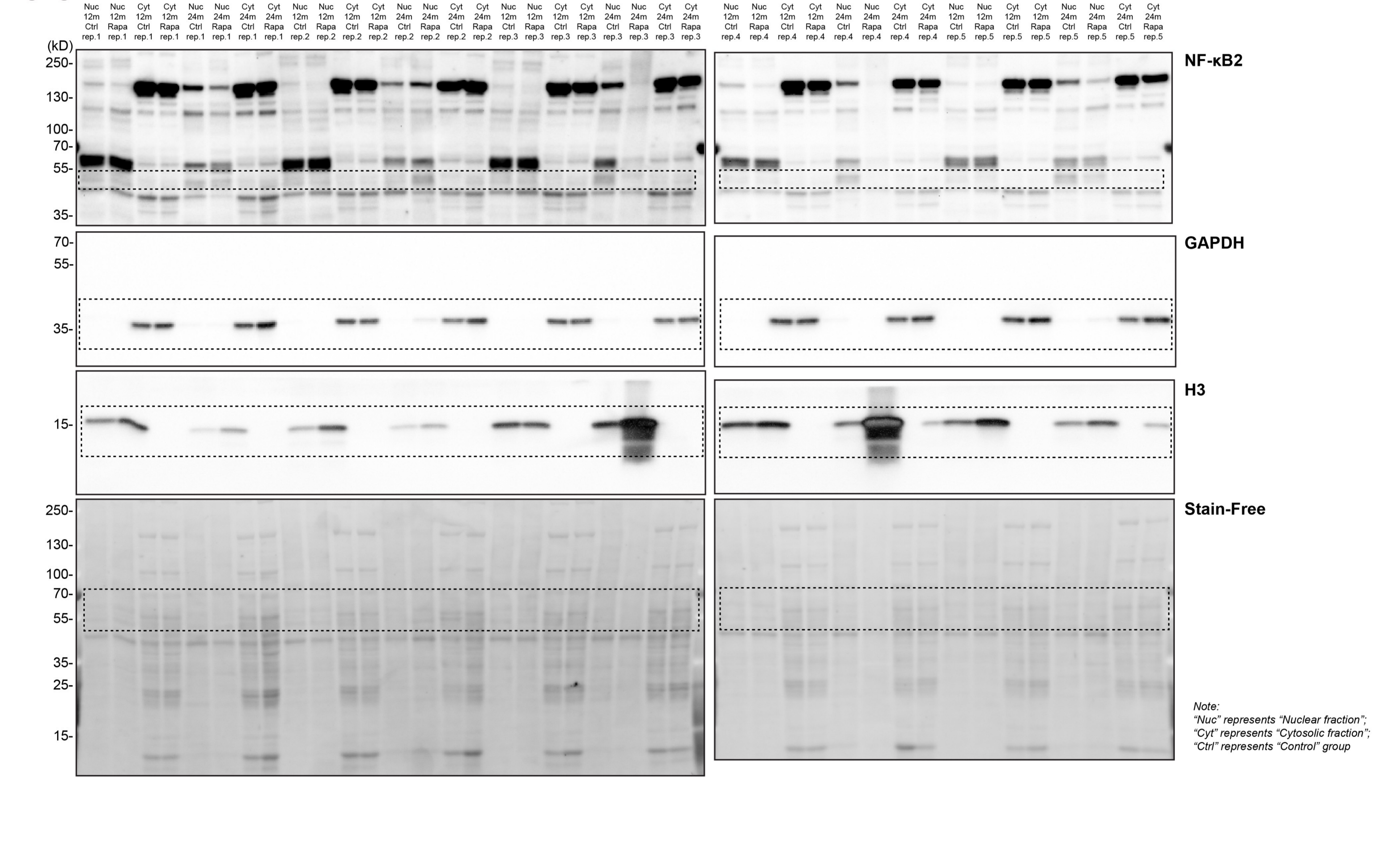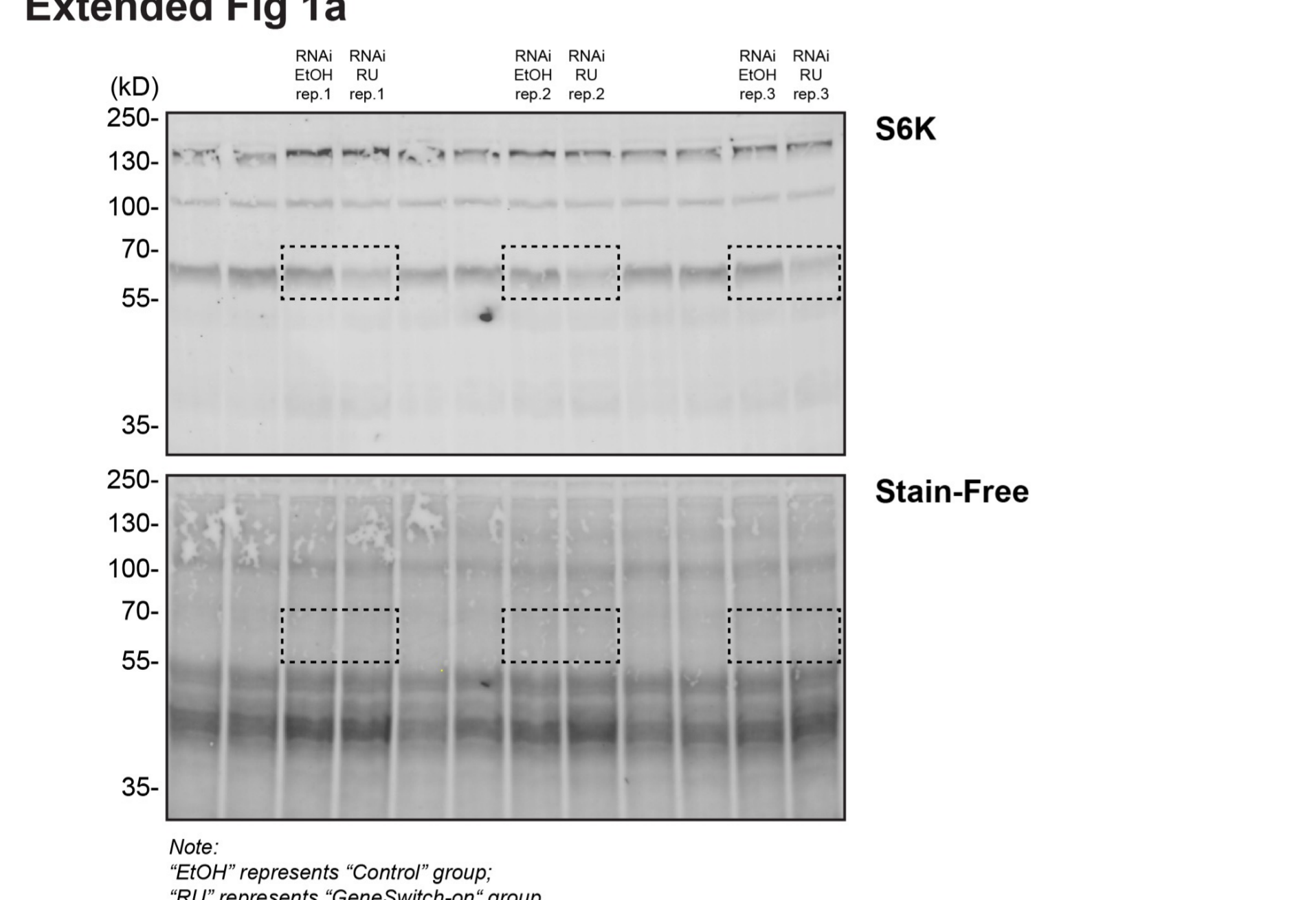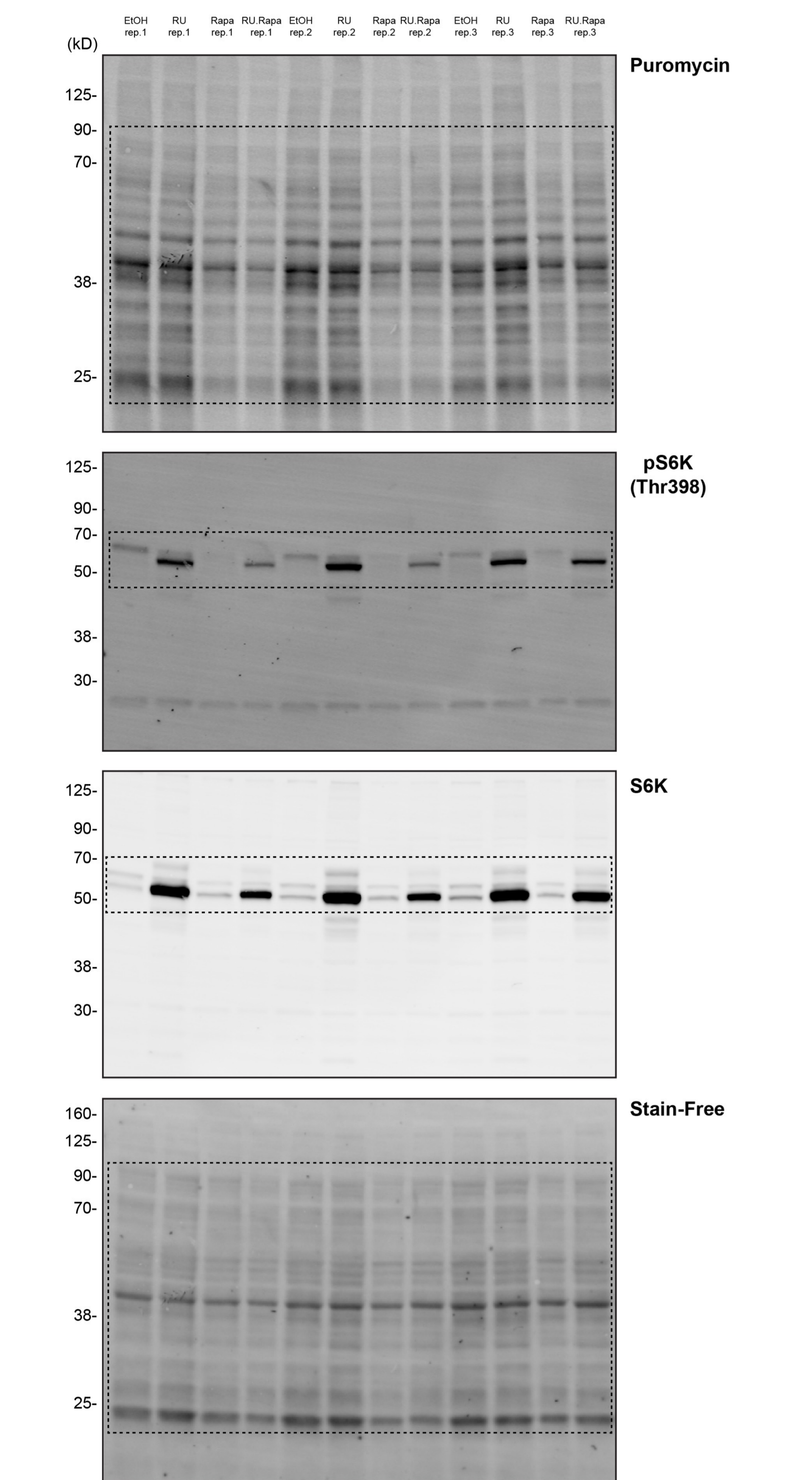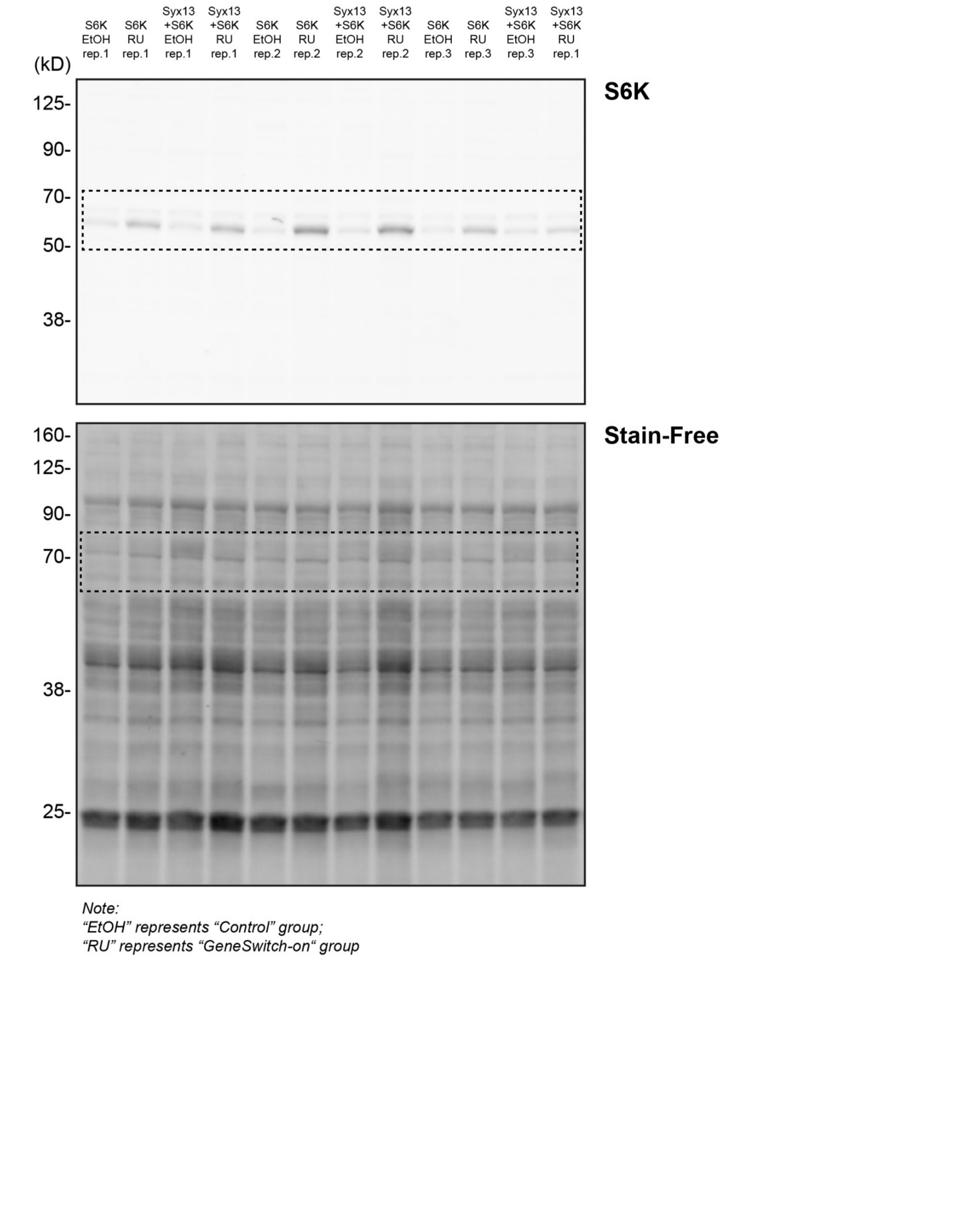

Supplement: Supplementary file 9 — A single PDF file containing all supporting blots, with clearly labeled blots for each Figure/Extended Figure item. [file 43587_2024_578_MOESM9_ESM.pdf]
